# The mitotic tensegrity guardian tau protects mammary epithelia from katanin-like1-induced aneuploidy

**DOI:** 10.18632/oncotarget.10728

**Published:** 2016-07-20

**Authors:** Haruka Sudo, Kazunori Nakajima

**Affiliations:** ^1^ Department of Biochemistry, The Nippon Dental University School of Life Dentistry at Tokyo, Chiyoda-ku, Tokyo 102-8159, Japan; ^2^ Department of Anatomy, Keio University School of Medicine, Shinjuku-ku, Tokyo 160-8582, Japan

**Keywords:** breast cancer, chromosome instability, microtubule severing, mitotic spindle, tau

## Abstract

The microtubule associated-protein tau has been identified as an effective positive prognostic indicator in breast cancer. To explore the physiological function of tau in early carcinogenesis, endogenous tau was knocked down in primary cultured human mammary epithelial cells. This resulted in chromosome-bridging during anaphase followed by micronucleation, both of which were suppressed by a further katanin-like1 knockdown. We also detected that the exogenously expressed katanin-like1 induction of cellular transformation is prevented by exogenous tau in rat fibroblasts. The mutant katanin-like1 (L123V) identified in breast cancer showed an increase in this transformation capacity as well as microtubule severing activity resistant to tau. The tau knockdown resulted in a loss of the kinetochore fibers on which tau is normally localized. This physical fragility was also observed in isolated tau-knockdown mitotic spindles, supporting the relevance of microtubule damage to the onset of transformation. The karyotyping of tau-knockdown cells showed increased frequency of loss of one X chromosome, further suggesting the involvement of tau in breast tumorigenesis. We propose that tau may contribute to tumor progression by protecting spindle microtubules from excess severing by katanin-like1. We also present data indicating that the microtubule-binding octapeptide NAP is a candidate modifier against the tau deficiency in tumor cells.

## INTRODUCTION

Tau is a member of microtubule-associated proteins (MAPs) which commonly have conserved C-terminal microtubule binding domains (MTB) [1]. The N-terminus is known as the projection domain and contributes to homophilic interactions [2]. Various functions of tau have now been established including microtubule (MT) assembly and stabilization. In addition, tau functions as a spacer between adjacent MTs [3] to bundle or pack these structures. Physiologically tau works in neurons involved in axonogenesis and growth cone motility. Various regulatory mechanisms of tau are also known. Changes to tau occur during neuronal development including a shift from shorter fetal to longer adult isoforms and a decrease in phosphorylation [4]. The phosphorylation of tau has been considered to decrease its affinity for MTs.

With regard to tau phosphorylation, the growth cone, which is the most dynamic domain in axons, may provide a representative example for understanding phosphorylated-tau functions. In the growth cone, the binding of tau to MTs has been shown to be quite labile and not to contribute to the stabilization or increase in the MT polymer mass. Instead, it is suggested that the effects of MT packing through the intermolecular projection domains may function in this process [5]. Tau is phosphorylated in growth cone MTs in response to cues from the extracellular milieu, and is thereby mobilized to contribute to the reorganization of distally splaying MTs into a packed form [6]. Another example of this process is seen in mammalian corticogenesis. Radial neuronal migration is known to require dynamic MTs, in which tau phosphorylation by MARK2 is involved. The inhibition of MARK2 leads to the abnormal accumulation of multipolar migratory neurons [7, 8] at the boundary of the intermediate zone. Even successfully transformed bipolar migratory neurons show an abnormally curbed leading edge and barely reach the cortical plate [9]. Thus, it has been suggested that phosphorylated-tau is involved in the packing of MTs as well as maintaining their dynamic characteristics. A common molecular basis for the role of tau in dynamic MTs has been predicted, i.e. MT packing via the tau projection domain.

Yeast MHP1 protein which contains an amino acid sequence homologous to the MTB of tau is associated with the organization of mitotic spindles [10]. In mammals, tau is also expressed in extra neuronal tissues [11]. Alzheimer's disease has a well-known pathology, the “neurofibrillary tangle”, which led to the discovery of abnormally phosphorylated tau. It has been proposed that mitotic tau phosphorylation mimics the hyperphosphorylated-tau state in Alzheimer's disease [12].

In a previous randomized clinical trial, tau was unexpectedly found to be an indicator of a good prognosis [13]. There have been a number of studies also indicating that tau is a positive prognostic factor in breast cancer [14–20]. In the Human Protein Atlas (http://www.proteinatlas.org/) [21] database, more than 50% of the cases with ductal carcinoma of the breast show a decrease in tau expression, with the remaining cases showing no tau level changes.

The katanin family comprises MT severing proteins with a conserved C-terminal AAA ATPase domain. These proteins form hexameric rings on the MT lattice [22–24] and ATP hydrolysis induces lateral defects in the local MT wall and, finally, MT breakage with two closely opposed ends remaining [25]. Functional analyses have revealed diverse roles for these severing proteins in mitosis and meiosis [25, 26], and in neuronal morphogenesis [27, 28]. We have previously analyzed the mitotic functions of katanin associated with the tumor suppressor LZTS2/LAPSER1 [29, 30].

Additional katanin p60-like proteins have been reported (KATNALs) [31, 32]. There have also been two major functional studies of mammalian KATNAL1 (KL1) [31, 33]. The first of these established the functions of KL1 in the mitotic spindle and found that during mitosis the localization of KL1 is restricted to spindle poles but is absent from the centrosomes [31]. KL1 was further shown in that study to increase MT density at spindle pole areas. The second of these reports established that KL1 has a role in spermatogenesis [33]. A mutation in *Katnal1* causing a loss of severing was identified as a cause of male specific infertility in the mouse. The Human Protein Atlas indicates low levels of KL1 protein expression in normal breast tissue [21]. In this database, about 50% of the cases with ductal carcinoma of the breast show an increase in the expression of KL1. In addition, none of the examined breast ductal carcinoma cases in the Human Protein Atlas database show any increase in either katanin p60 or KL2 expression.

A number of studies have reported that chromosomal instability (CIN) [34, 35] not only correlates with tumorigenesis, but may in fact be an initiator of this process [36]. Four direct mechanisms of CIN are known: 1) chromosome (Chr) cohesion defects, 2) spindle assembly checkpoint (SAC) defects, 3) supernumeral centrosomes, and 4) defects in kinetochore (Kt)-MT dynamics. Studies of Kt-MT attachments have so far focussed mainly on the molecules surrounding the Kt [37]. On the other hand, the involvement of MT binding molecules which do not directly associate with Kt has mostly been left unexplored.

Although tau localization at the mitotic spindle has been described [38], its physiological function is largely unknown. It has been reported that aneuploidy is induced in *tau* knockout mice [39]. In addition, familial tauopathy hereditary frontotemporal dementia with parkinsonism linked to chromosome 17 (FTDP-17) patients have been reported to have aneuploidy [38, 40]. Tau inhibits katanin in neurons [28, 41–43] and we have observed in fibroblasts that the Alzheimer's disease-related pseudo-hyperphosphorylated (PHP)-tau and FTDP-17-derived mutant tau have a reduced capacity for this inhibition [44]. In parallel, however, we also found that PHP tau still had significant inhibitory properties towards katanin [44].

In our current study, we hypothesized based on the cumulative evidence to date that the tau localized at the mitotic spindles has a protective function against KL1 and we explored the relevance of this phenomenon to early breast carcinogenesis.

## RESULTS

### Tau protein expression in human breast tumor tissues

**Figure 1:**
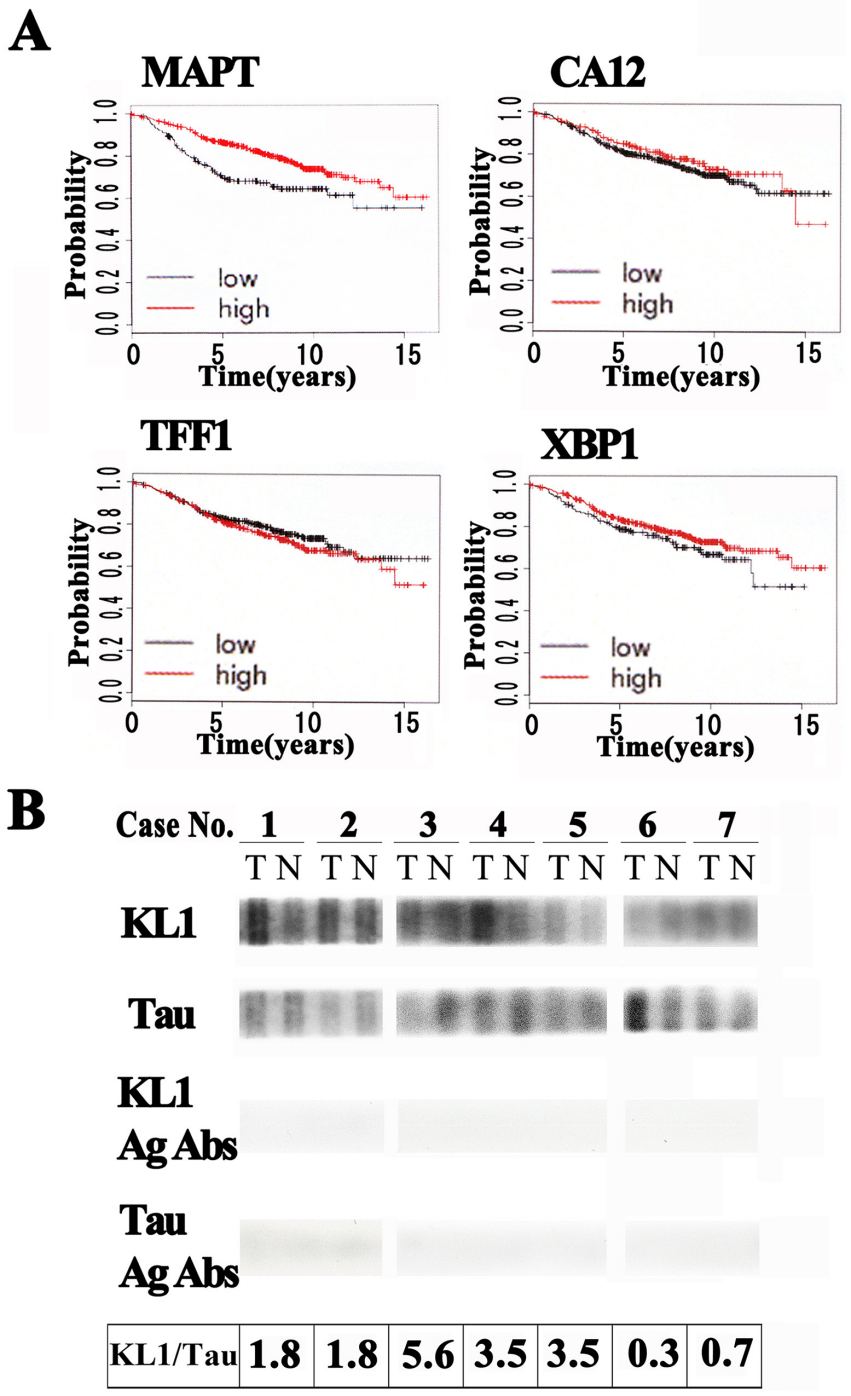
Tau protein expression in human breast tumor tissues **A.** Kaplan-Meyer analysis of up-regulated ERα target genes against ERα-positive breast tumors. The patient group with high tau expression (MAPT) showed a higher recurrence free survival probability compared with the low expression group. Other known up-regulated estrogen target genes (CA12, TFF1, and XBP1) did not show significant associations. **B.** Human female breast cancer tumor (T)/normal (N) tissue lysate test strip arrays immunoblotted with anti-KL1 (KL1), anti-total tau (Tau) or antigen absorbed antibodies (KL1 Ag Abs and Tau Ag Abs). KL1/Tau ratios (the relative expression of KL1 in tumor cells / the relative expression of tau in tumor cells) are presented in the bottom panel.

Using an online database that integrates multiple breast cancer datasets [45], we examined the prognostic properties of tau and found it to be an effective indicator of a good prognosis consistent with previous studies [14–20]. Tau is an up-regulated primary target gene of estrogen receptor and this makes the interpretation of clinical statistics complex because the estrogen receptor (ERα) positivity predicts a better endocrine therapy effectiveness. To gain further insight into these pathways, we examined the association of tau and three known up-regulated primary ERα target genes with prognosis [46] (Figure 1A, see Materials and Methods). The patient group with high tau expression showed a significantly better prognosis, but this was not evident for the other three estrogen target genes. To exclude the possibility that the higher tau levels might simply reflect a higher ERα expression, we further stratified our patients based on their ERα expression levels. The results showed no significance however. These findings led us to suspect that tau has some physiological functions in breast tissue.

KL1 has been suspected to be inhibited by tau [31]. With this in mind, we tested its relative expression levels in primary human breast cancers. We immunoblotted human breast cancer tumor/normal tissue lysate strips from seven patients (cases 1 to 7) with both anti-KL1 and anti-tau antibodies. The detected signals on these strips were lost when the antibodies were pre-absorbed with antigens (Figure 1B lower panels), supporting the specificity of the signals. In the tau blots, the relative ratios of the tumor sample to control signals were 1.04 ± 0.11, 0.51 ± 0.03, 0.18 ± 0.01, 0.60 ± 0.03, 0.82 ± 0.03, 1.92 ± 0.13, and 1.30 ± 0.06 (mean signal intensity ± S.D.; n=3) for cases 1 to 7, respectively. The corresponding ratios in the KL1 blots were 1.90 ± 0.10, 0.92 ± 0.05, 1.00 ± 0.07, 2.10 ± 0.20, 2.83 ± 0.45, 0.56 ± 0.05, and 0.97 ± 0.07 (n=3), respectively. When we calculated the KL1/tau ratios (Figure 1B) five of these cases showed an increase, one a decrease, and one patient showed no change. With respect to a possible stratification-based future treatment strategy for these patients, most attention would need to be paid to case 3, who showed a remarkably high KL1/tau ratio of 5.6. We next determined what would be evoked in cultured cells under a high KL1/tau ratio.

### Transformation studies in RFL6 cells

Rodent fibroblast lines have been the established tester cells in prior transformation studies. We used the rat fibroblast cell line RFL6 as its flat morphology facilitates MT status and transformation analysis in the same cell. In our focus formation experiments, because transfection of the authentic oncogenes H-Ras (G12V) and ErbB2 (V659E) [47] (Supplementary Figure S1) resulted in few colonies, we performed a selection and obtained 15.2 ± 0.7 focus-forming units (FFU) (average ± S.D.; n=4) and 11.8 ± 2.1 FFU, for Ras and ErbB2 mutants, respectively.

**Figure 2:**
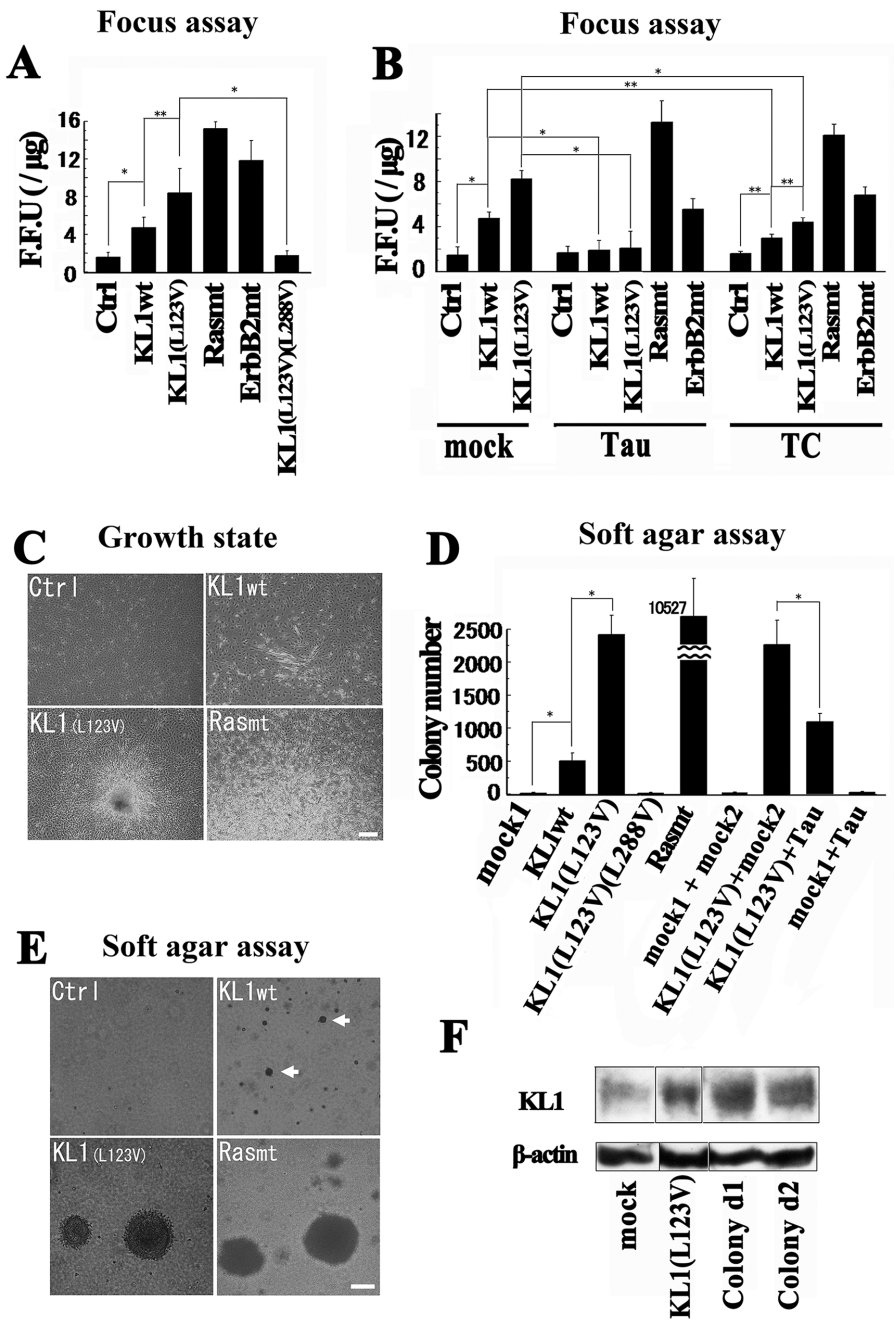
Transformation studies in RFL6 cells **A.** RFL-6 cells were transfected with wild-type KL1 (KL1wt), KL1 (L123V), oncogenic Ras (Rasmt), oncogenic ErbB2 (ErbB2mt), or KL1(L123V)(L288V). The transformation efficiency was measured by the number of FFU (*n* = 4). **B.** Focus assay of stable tau or TC expressing cells. (*n* = 3) **C.** Morphological and growth characteristics of stable KL1 expressing cells growing in monolayers. Empty vector control (Ctrl), KL1wt, KL1(L123V), and Rasmt stable lines were established. The Rasmt line showed spontaneous formation of larger foci which consisted of refractile and fusiform cells in criss-crossed arrays (Rasmt: lower part). KL1(L123V) expressers showed spontaneous foci formation with massive piling up into virtually opaque multilayers. KL1wt cells showed heterogeneity; altered slender and long cells appeared. Bar, 200 μm. **D.** KL1wt, KL1(L123V), KL1(L123V)(L288V), KL1(L123V) and Tau, Tau, and Rasmt stable lines were plated in soft agar and macroscopic colonies were counted (*n* = 3). **E.** KL1wt cells showed small-sized colony formation. KL1(L123V) induced colonies comparable to those by Rasmt in size. Bar, 100 μm. Arrows indicate the small-sized colonies. **F.** KL1 protein expression in KL1(L123V)-induced colony-derived cells (Colony d1 and d2) was analyzed by western blot. β-actin was used as a loading control. The asterisks and double asterisks indicate significant differences (Student's *t*-test, *P*< 0.01 and *P*< 0.05, respectively).

We included a mutant KL1 (L123V) identified by a previous study of gene mutations in human breast cancer [48] in addition to human wild-type KL1 (KL1wt) in these transformation experiments. First, we observed significant foci formation in KL1wt expressing cells compared with the controls (Figure 2A). KL1(L123V) expressers generated twice the number of foci. The MT severing of KL1 has been found to be abolished when an L288V mutation is introduced into its ATPase domain [33]. A double mutant KL1 containing both L123V and L288V substitutions showed no significant foci formation. We also established stable cell lines of RFL6 cells expressing the full-length tau that possesses two N-terminal insertions, the C-terminal exon10-derived repeat domain (2N4R; 441 amino acids) and tau C-terminus fragment (Supplementary Figure S1) (TC; Supplementary Figure S2). TC lacks the projection domain but includes the MTB. We performed similar analyses with these constructs and found that full-length tau expressers almost completely inhibited foci formation by KL1wt or KL1(L123V) and even TC expressers showed smaller but significant inhibition of foci formation by these stable transformants (Figure 2B).

We next performed soft agar assays for which we established KL1wt and KL1(L123V) stable expressers. In liquid culture, as shown in Figure 2C, the KL1(L123V) expressers formed spontaneous foci in a comparable manner to Ras mutant stable expressers whilst the KL1wt expressers showed heterogeneity in their morphologies. In the assay, the Ras mutant expressers showed efficient colony formation, whereas few colonies were formed in the control. Strikingly, the KL1(L123V) expressers also showed significant colony formation (Figure 2D and 2E), as did the KL1wt expressing cells although of smaller size and number. The stable KL1(L123V)(L288V) expressers showed no significant colony formation. Furthermore, the cells that stably co-expressed KL1(L123V) and full-length tau showed significant foci suppression compared with the KL1(L123V) expressers (Figure 2D). These findings suggest that the driving force behind the transformation by KL1 originates from its enzymatic activities which are inhibited by tau via its interaction with MTs.

The colonies were isolated from agarose gel containing KL1(L123V) expressing cells and assessed for protein expression. We found a retained exogenous protein level (Figure 2F) thus supporting the contribution of KL1(L123V) to the transformation.

### Biological properties of KL1 expressers

**Figure 3:**
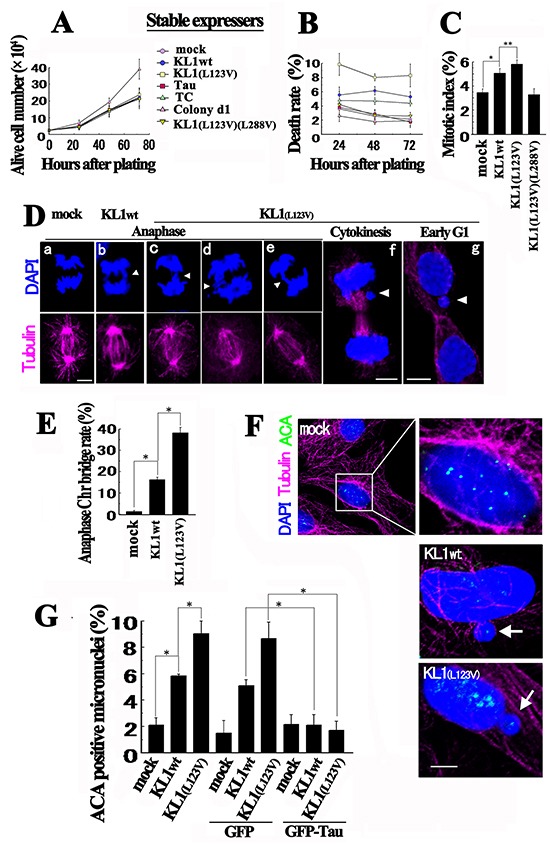
Biological properties of KL1 expressers **A.** Equivalent numbers of RFL6 cells stably expressing control vector (mock), KL1wt, KL1(L123V), Tau, TC, and KL1(L123V)(L288V), and Colony d1 cells, were plated. Cells negatively stained by trypan blue were counted at the indicated time points (*n*=3). **B.** The same cultured cells described in (A) were monitored for trypan blue positivity (indicating dead cells) (*n*=3). **C.** Cells in (A) were analyzed to determine the mitotic index (>100 cells were counted, *n*=3). **D.** During anaphase, Chr bridges were detected in KL1 expressing lines by staining for tubulin and DNA. The major pattern is indicated in panels b and e; the minor complex patterns are shown in panels c and d. Lagged Chrs (f, arrow head) and micronuclei (g, arrowhead) were also observed in the later phases. **E.** Quantification of Chr bridges. The number of Chr bridges/ number of total anaphases was determined (>100 anaphases were counted; *n*=3). **F.** Micronucleation in KL1 expressing cells. Cells were stained for tubulin, centromeres (using ACAs), and DNA. In control cells, ACA signals were detectable in the nucleus (enlarged view). Most of the micronuclei (~90%) showed ACA signals (arrows). **G.** Quantification of ACA positive micronuclei in stable KL1 expressers under transient GFP-tau expression (>200 interphase cells were counted, *n*=3). Bars, 5μm. The asterisks and double asterisks indicate significant differences (Student's *t*-test, *P*< 0.01 and *P*< 0.05, respectively).

To determine the biological activities that are causal for the transformation by KL1, we first analyzed cell proliferation and cell death in liquid culture. Compared with the controls, stable KL1 expressers showed only a slight increase in cell death and no change in proliferation (Figure 3A and 3B). KL1(L123V)(L288V) cells showed no change and the stable tau (2N4R or TC) expressers showed no or only marginal changes. These data suggest that KL1 does not provide simple growth advantages. We then evaluated the mitotic index and detected small but significant increase in the KL1 expressers (Figure 3C). We thus analyzed the morphologies of these mitotic cells by immunostaining. Prior to anaphase, we could not detect any aberrations, but during anaphase, we detected Chr bridge formation (Figure 3D). Quantitatively, the frequencies of Chr bridges were 16% and 38% in KL1wt and KL1(L123V) expressing cells, respectively (Figure 3E). By contrast, the control cells showed only a 1.5% rate of Chr bridge formation. In later phases we observed lagging Chrs and micronuclei (Figure 3Df and 3Dg). These phenotypes were also observed in cells that were transiently transfected with KL1 variants (Supplementary Figure S3).

These results indicated a triad nature, the absence of proliferative promotion, and slightly increased cell death and mitotic index, characteristics which have been reported previously in the process of transformation driven by CIN [36, 49–51]. The correlation between the frequency of Chr bridges and the magnitude of the transformation also suggests the involvement of CIN. We therefore next analyzed the state of the nuclei in interphase cells. Immunostaining with the anti-centromere antibodies (ACA) detected whole Chr micronucleation [52] in KL1 expressers (Figure 3F), which was almost completely inhibited by the transient expression of GFP-tau (Figure 3G). These data led us to hypothesize that KL1 induced transformation is through CIN.

### Tau protects MTs from KL1-induced MT severing in RFL6 cells

To clarify whether tau inhibits KL1, we performed RFL6-based MT sensitivity tests as described previously [43, 44]. We tested multiple forms of human tau constructs, which included the adult form of tau (2N4R), a much longer tau variant (Big; 833 amino acids) [11], the shortest embryonic tau variant (0N3R; 352 amino acids), TC, and PHP. In addition to the utility of the PHP construct for in-depth analyses of mitotic phosphorylation, we newly constructed a pseudo mitotically phosphorylated-tau (MHP) by substituting eight amino acids (T153, T181, S202, T205, T212, S214, T217, and T235) with glutamates, based on a previous study [12] (Supplementary Figures S2 and S4).

**Figure 4:**
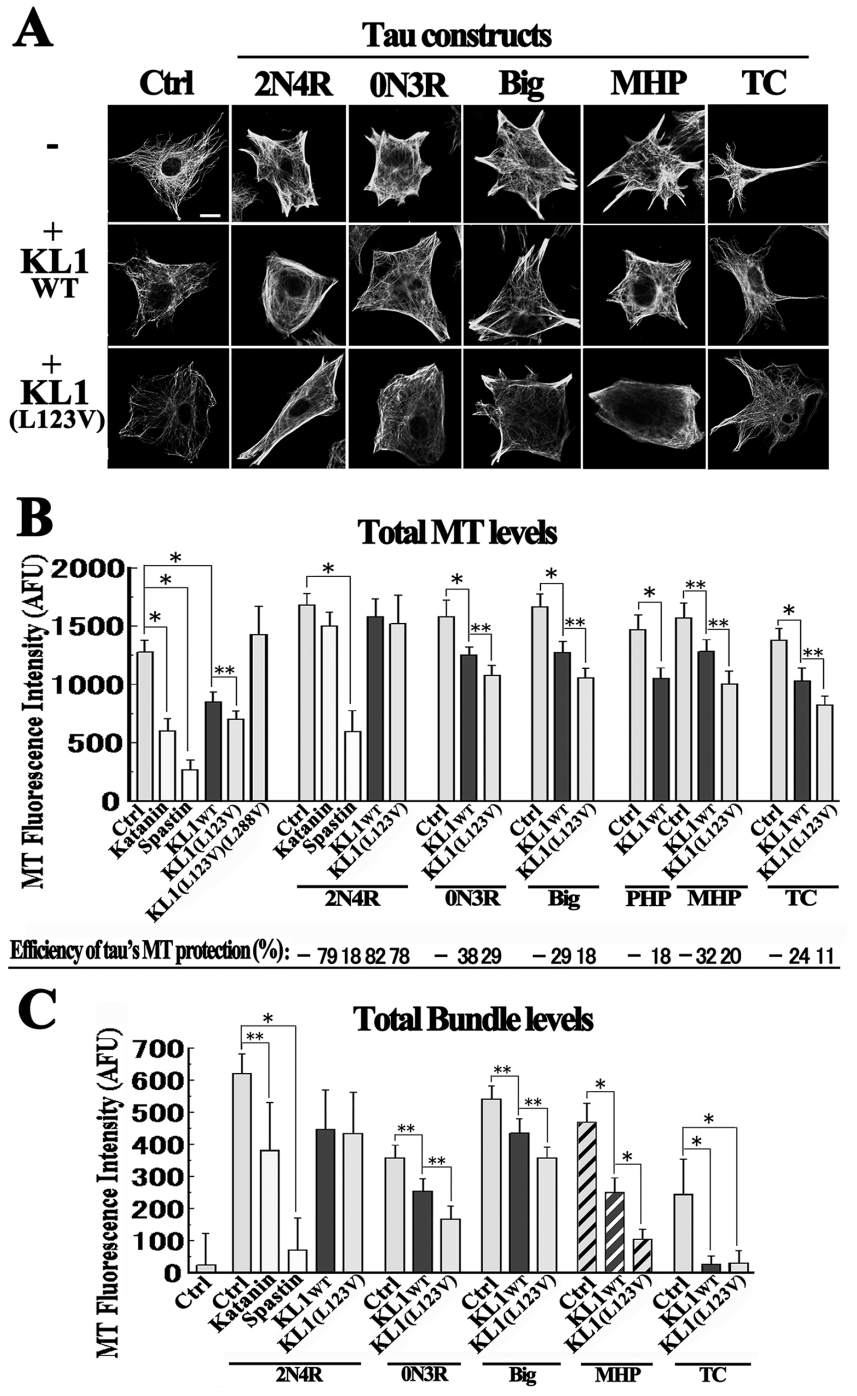
Tau protects MTs from KL1-induced MT severing in RFL6 cells **A.** Representative images showing the effects of the indicated tau constructs on KL1-induced MT severing in RFL6 cells. Upper row: cells were transiently transfected with tau constructs and stained for tubulin including non-expressing control (Ctrl) cells and cells expressing 2N4R, 0N3R, big tau (Big), mitotic pseudo-phosphorylated-tau (MHP), and tau C-terminus (TC). Middle and lower rows: cells were co-transfected with one of the tau constructs together with either KL1wt or KL1(L123V); bar, 10 μm. **B.** Quantification of total MT levels. The efficiencies of MT protection by the different tau constructs are indicated below the bar graphs. **C.** Quantification of the total MT bundle levels. The largest difference between KL1wt and KL1(L123V) was observed in MHP co-expressing cells (shaded bars). AFU, arbitrary fluorescence unit. The asterisks and double asterisks indicate significant differences (Student's *t*-test, *P*< 0.01 and *P*< 0.05, respectively).

Figure 4A shows representative results of these analyses. Quantification was conducted as described previously [43, 44] (Figure 4B). Compared with the controls, the 2N4R expressers showed higher total levels of MTs and of MT bundling as observed previously [44]. Cells expressing 0N3R or PHP showed weaker MT phenotypes, also as reported previously [41, 44] whereas cells expressing Big or MHP displayed intermediate effects. TC produced the least effects on the MTs. In terms of total MT levels, katanin and spastin overexpression induced large reductions (59% and 79%, respectively) and KL1wt overexpression induced a 34% reduction consistent with previous studies [31, 33]. KL1(L123V) overexpression induced a significantly higher MT reduction (45%) whereas KL1(L123V)(L288V) had no effect.

When katanin and spastin were co-expressed with 2N4R, an MT reduction trend (11%) that was non-significant was detected in katanin+2N4R cells, whilst a much greater reduction (65%) was detected in spastin+2N4R cells, consistent with previous reports [28, 44]. In 2N4R+KL1wt cells, the MT reduction was also not significant, suggesting that KL1 is similar to katanin. When KL1wt was overexpressed in cells expressing 0N3R, Big, or MHP, inhibition of the MT reduction was evident but was not as pronounced as that seen with 2N4R. With regard to the TC product, an MT reduction was prominent but still showed some degree of inhibition. Again, KL1(L123V) showed a tendency to induce more reduction than KL1wt in combination with these tau constructs.

The inhibitory effects of tau were standardized by severing activities, and the protection efficiencies of 2N4R against katanin or spastin were 79% and 18%, respectively. Against KL1wt, the protection efficiencies of 2N4R, 0N3R and Big were 82%, 38% and 29%, respectively, whereas those of PHP and MHP were 18% and 32%, respectively (Figure 4B), further indicating a moderate degree of inhibition by MHP. In terms of total bundle levels, we found the largest difference between KL1wt+MHP and KL1(L123V)+MHP cells (47% and 78% reductions, respectively; Figure 4C shaded bars) among all comparisons of KL1 variants, suggesting that MT bundles are more sensitive to the altered severing activity of KL1(L123V).

### Characterization of HMECs

**Figure 5:**
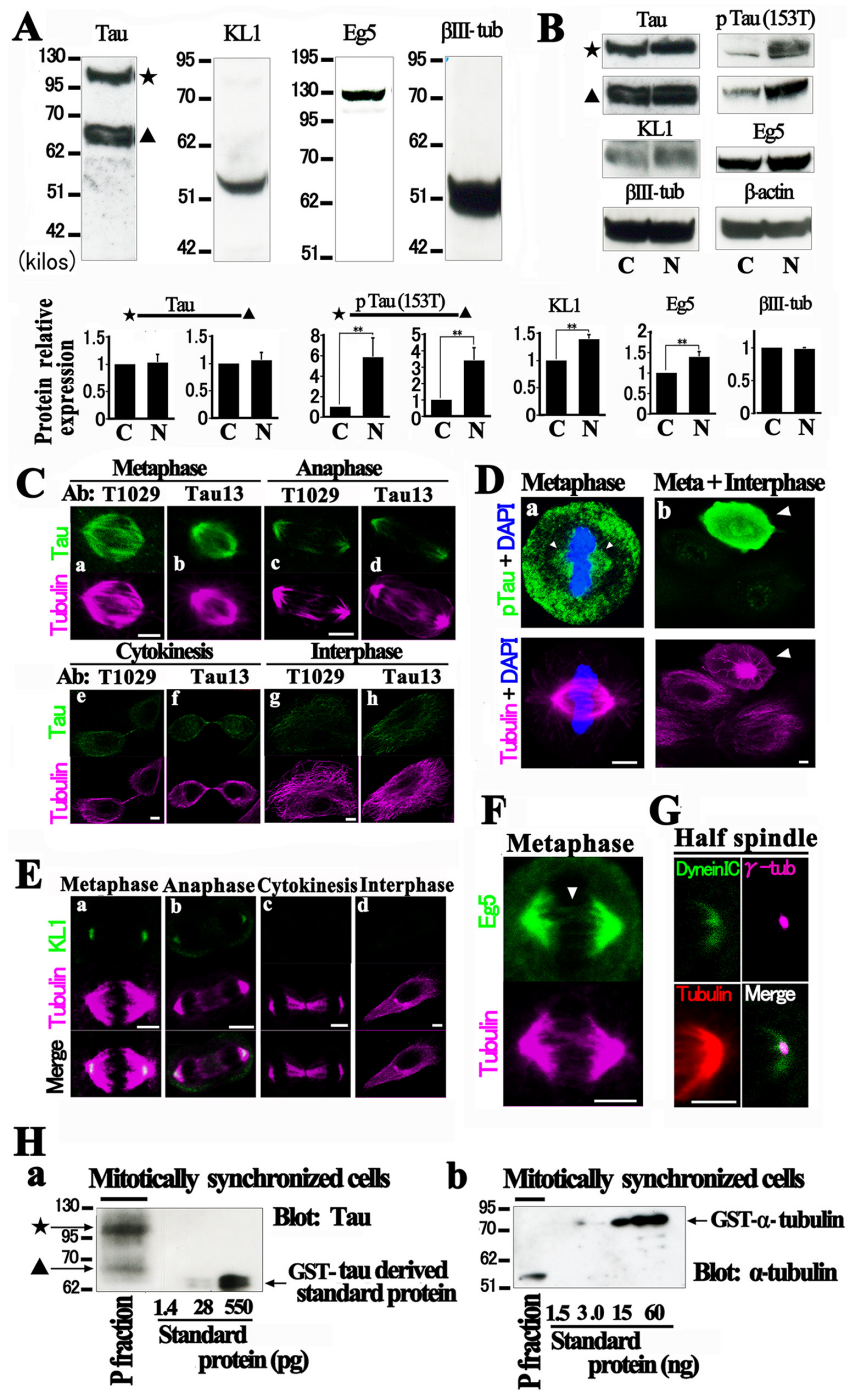
Characterization of HMECs **A.** Western blotting analysis of tau, KL1, Eg5, and neuron specific beta-III tubulin (βIII-tub) in HMECs. The star denotes big tau and the triangle indicates the normal tau species (Tau). **B.** Mitotically synchronized cells following nocodazole treatment (N) were comparatively analyzed against unsynchronized control cells (C) for the expression of the same proteins in (A) and also phospho-tau (153T) (pTau (153T)). The control tau and Eg5 bands are as decsribed in (A). The bar graphs show quantification of the protein expression (*n*=3). The ratios of mitotic protein expression to the control are shown. The expression of protein was normalized to β-actin protein expression. The double asterisks indicate significant differences (Student's *t*-test, *P*< 0.05). (**C** and **D)** HMECs were immunostained with anti-total (T1029 and Tau13) and phospho-tau (153T) antibodies (green). **C.** During metaphase and anaphase, tau was found to localize in the spindle. **D.** Phospho-tau spindle localization (arrowheads) was also detected (a). Mitotic cells (arrowhead) showed stronger signals than interphase cells (b). **E.** Immunostaining with anti-KL1 (green) antibody detecting a polar KL1 distribution in metaphase and anaphase (a and b). **F.** Immunostaining for Eg5 (green). In addition to a major broad half spindle distribution of Eg5, “static Eg5” (arrowhead) was detected in the middle of the spindle. **G.** Immunostaining for cytoplasmic dynein (green), γ-tubulin (magenta), and tubulin (red). **H.** Molar ratio of tau to tubulin heterodimers on the mitotic spindle. Polymerized MTs (P fraction) were purified from mitotic HMECs and assessed for total tau and tubulin by immunoblotting. GST-tagged proteins were used as standards. Bars, 5μm.

Most human breast cancers originate from mammary epithelial cells (HMECs). To address whether our observations in rat cells were not simply species- or cell type-specific effects [53], we analyzed HMECs and focused on their Chr status. First, we examined the endogenous protein expression profiles in these cells. As indicated in Figure 5A, a total-tau immunoblot detected two major bands at about 68 kilo dalton and 110 kilo dalton. The former may correspond to normal tau [54] and the latter to the big tau variant. Neither tau species showed any change when mitotic cells were enriched (Figure 5B). KL1 was detected at 55 kilo dalton and showed a moderate but significant increase in the mitotic cells as well as mitotic kinesin Eg5, whereas neuron-specific beta-III tubulin [55] did not show any change. The relative expression ratios to the control were 1.39 ± 0.08 and 1.39 ± 0.13 for KL1 and Eg5, respectively (n=3, p<0.05 for both proteins). Among the mitotic phosphorylation sites on tau [12] we chose 153T because it seemed to be the most phase-specific. Western blot analysis with a phopspho-153T specific anti-tau antibody detected significantly more phosphorylation on both the normal and big tau species during mitosis (Figure 5B). The relative expression ratios to the control were 3.40 ± 0.78 and 5.84 ± 1.9 for normal and big tau products, respectively (n=3, p<0.05 for both).

Immunostaining studies with two anti-total tau antibodies against different epitopes detected localization on the mitotic spindle in both metaphase and anaphase (Figure 5C) but much weaker tau signals in later phases. Immunostaining with anti-phospho-153T tau antibody also detected the interaction of phospho-tau with the spindle (Figure 5D). Intense phospho-tau signals were observed specifically in mitotic cells, verifying their specificity [12] (Figure 5D). These data support our hypothesis that tau still binds to the MTs during mitosis. In KL1 immunostaining analysis, we detected polar localization of KL1 in metaphase and anaphase and that these signals also became much weaker in later phases (Figure 5E), as previously reported [31]. These data suggest that KL1 also severs polar region MTs in the non-cancerous HMECs as effectively as previously reported in a cancer cell line [31].

Eg5 staining detected weak signals on the overlapped zone of interpolar MTs, which has been shown to be static [56], in addition to the major half spindle-localized signals (Figure 5F). Cytoplasmic dynein is known to cross-link MT minus ends in the spindle poles [57]. In contrast to KL1 [31], we found that the γ-tubulin signals were entirely embedded in the dynein signals (Figure 5G). To confirm the binding of tau to the spindle MTs, we adopted a biochemical approach [58]. Mitotic synchronized cells were collected by treatment with MG132 [59], and the polymerized tubulin was purified, in which we detected tau (Figure 5H). The relative molar ratio of tau to the tubulin heterodimer was assessed and found to be roughly 1/225 and 1/194 for big and normal tau, respectively. These are higher than the 1/236 ratio at which tau has been reported previously to have suppressive effects on MT dynamics [60].

### Functions of tau in Kt fibers

**Figure 6:**
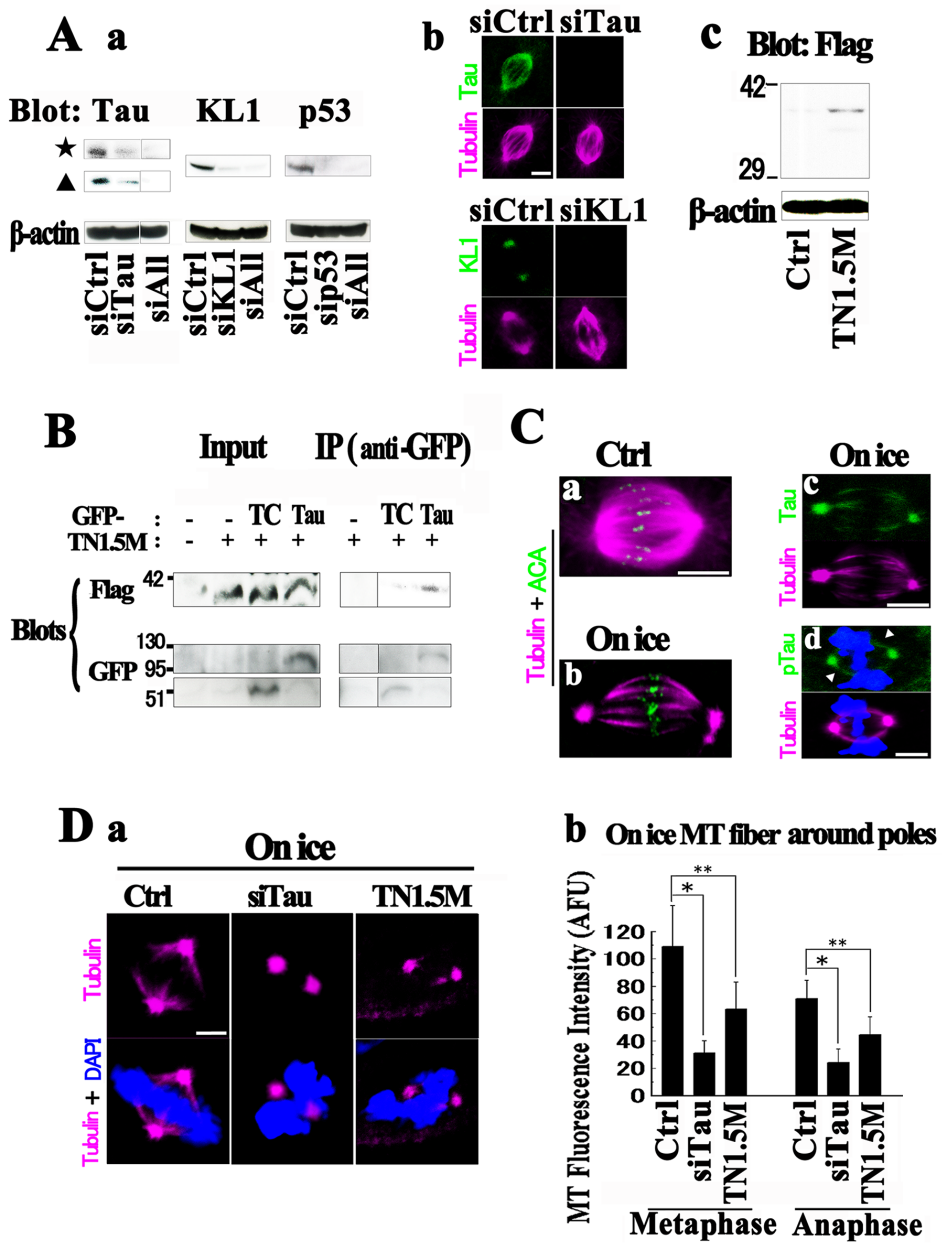
Functions of tau on Kt fibers **A.** (a) HMECs were transfected with siRNAs against tau (siTau), KL1 (siKL1), p53 (sip53), or a combination of all three (siALL). An approximately 90% reduction in the level of each protein was achieved. (b) siRNA-transfected HMECs were stained for the indicated proteins. (c) Expression of transfected TN1.5M in HMECs. **B.** RFL6 cells were transfected with GFP-TC, GFP-Tau, and TN1.5M constructs. Immunoprecipitates (IP) from GFP-Tau+TN1.5M cells with anti-GFP yielded significantly more TN1.5M than those from GFP-TC+TN1.5M. The input lane contained total lysates as a control. **C.** HMECs were cold treated and co-stained with tubulin (magenta) and ACA (green), or with total tau (Tau) or anti-phospho-tau (153T) (pTau) antibodies. The cold treatment left only Kt fibers behind (On ice; b) and polar tubulin accumulation was observed. The localization of tau (c) and phospho-tau (arrowheads in d) on Kt fibers was detected. **D.** Tau-KD or TN1.5M expressing cells under cold treatment were analyzed for MTs. (a) Significant reductions in Kt fibers were detected in both cells. (b) Quantification of the Kt fibers in (a). Both prometa/metaphase (Metaphase) and anaphase (Anaphase) were analyzed (>40 cells were counted, *n*=3). Bars, 5μm. The asterisks and double asterisks indicate significant differences (Student's *t*-test, *P*< 0.01 and *P*< 0.05, respectively).

Neurology studies have established that tau protein functions in the bundling of MTs. The Kt fibers are also MT bundles [61–63] and so we assessed the function of tau in the biology of these structures. We adopted two strategies to examine the function of tau in Kt fibers, tau knockdown (KD) and overexpression of the projection domain. Regarding the latter approach, our rationale was that tau contributes to the interaction of MTs through homodimerization via their projection domain [2]. We attempted transient tau KD by siRNA in HMECs and confirmed close to 90% reductions (Figure 6Aa). Immunocytochemicalstaining with anti-total tau antibodies also confirmed these effects (Figure 6Ab). There were no clear morphological aberrations in tau-KD spindles. With regard to the latter strategy, we generated 6 constructs of the tau N terminus. By examining the suppressive effects on GFP-Tau (2N4R)-induced MT bundling in RFL6 cells, we selected one of these constructs (TN1.5M) that showed efficient inhibition (Supplementary Figures S4 and S5). We also confirmed the physical binding of TN1.5M to tau by immunoprecipitation (Figure 6B). Overexpression of TN1.5M in HMECs (Figure 6Ac) showed neither observable cell toxicity nor spindle malformation.

Cold treatment is known to be useful for analyzing Kt fibers because it allows only Kt fibers to remain and they are considered to retain their physiological properties [63]. Under cold treatment, we found in our present study that HMECs showed efficient depolymerization of spindle MTs other than Kt fibers and we detected both tau and phospho-tau on these structures (Figure 6C). We then knocked down tau or overexpressed TN1.5M, and analyzed Kt fibers by immunostaining following cold treatment. Surprisingly, compared with the control, the tau KD reduced the amount of Kt fibers by more than 70% in both metaphase and anaphase (Figure 6D). We also detected significant reductions in these fibers in TN1.5M-transfected cells. Given that tau does not contribute to cold stability itself [64], these findings suggest that tau is involved in the formation or maintenance of Kt fibers. Since the tau-KD spindles showed no abnormalities, we speculate that this role of tau may be compensated for by other proteins in a functionally redundant manner at physiological temperatures.

### Perturbation of Chr segregation during mitosis in HMECs

**Figure 7:**
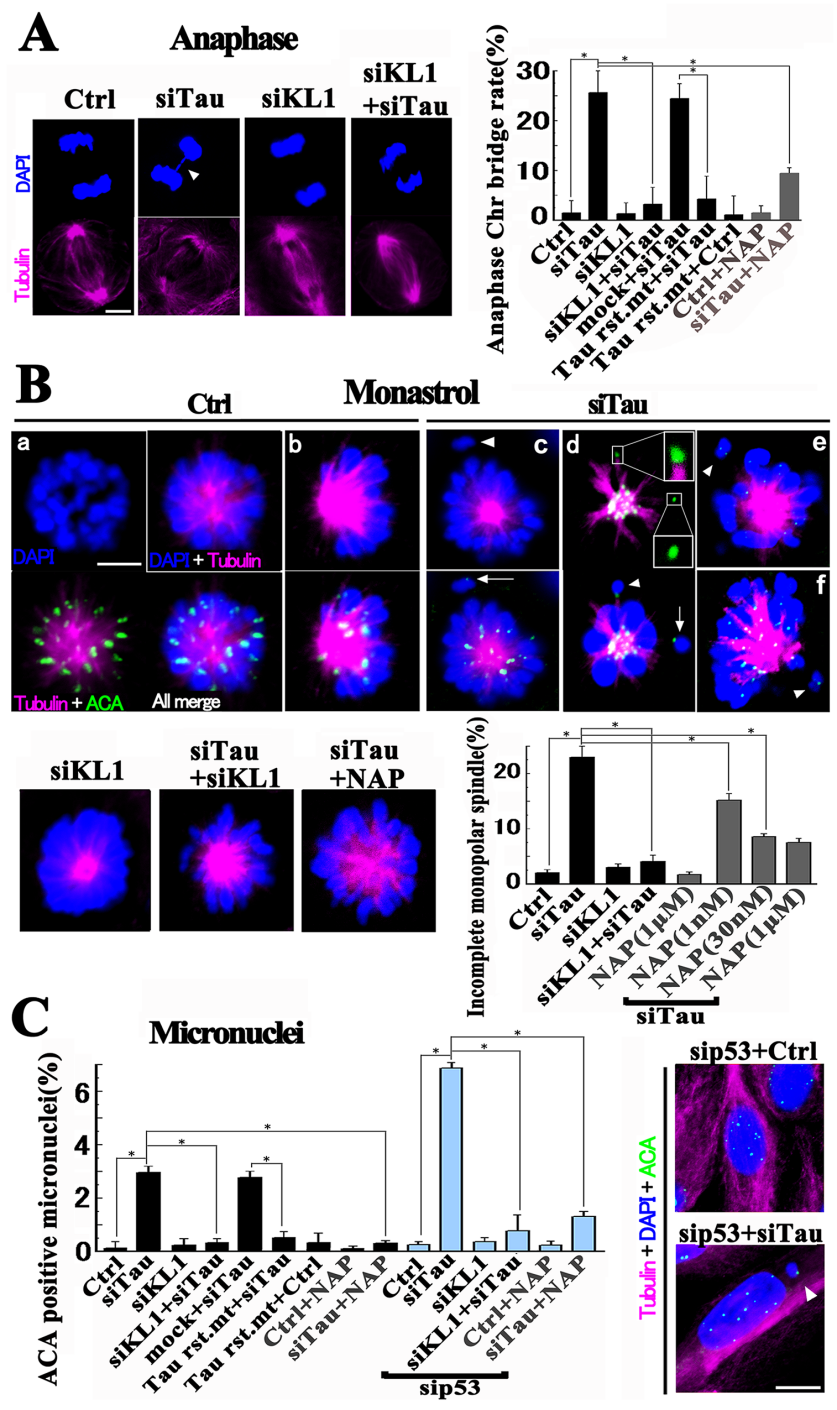
Perturbation of Chr segregation during mitosis in HMECs **A.** HMECs were knocked down for the indicated proteins and stained. Anaphase Chr-bridging in tau-KD cells (arrowhead in siTau) was inhibited by KL1 KD (siKL1+siTau). Bar, 5μm. The bar graph shows quantification of anaphase bridges (>40 cells were counted, *n*=3). Tau-KD resistant 2N4R (Tau rst. mt). **B.** Monopolar preparation by monastrol treatment. HMECs were knocked down for the indicated proteins with or without NAP, cultured, treated with monastrol for 5 hours, and stained. A rosette-like Chr alignment in the monopolar spindle was observed in control cells (Ctrl; a). Tau KD cells showed “incomplete” spindles (arrowheads in siTau c and e) with disconnected Chrs devoid of Kt MTs (arrow in siTau c). In some incomplete cases, disconnected Chrs with (upper inset) and without (lower inset) Kt MTs coexisted (siTau d). The minor (~5%) skewed spindles (Ctrl b (complete) and siTau f (incomplete)) were also analyzed. Both KL1-KD and 30 nM NAP treatment suppressed incomplete spindle formation. Bar, 5μm. The bar graph shows quantification of incomplete spindle formation (the number of incomplete spindles/total monopolar spindles) (>100 spindles were counted, *n*=3). **C.** HMECs treated with siRNAs or with 30 nM NAP were assessed for ACA positive micronucleation. The bar graph shows the quantification of the micronucleation (*n*=3). The asterisks indicate significant differences (Student's *t*-test, *P*< 0.01). The lower image shows the representative tau+p53 KD cell with an ACA positive micronucleus (arrowhead). Bar, 10 μm.

Based on our finding that excess KL1 activity induced micronucleation and transformation in RFL6 cells which was suppressed by tau, we examined the effects of an increased KL1/tau protein ratio on Chr segregation in HMECs through the knockdown of tau, KL1, or both. An efficient (near 90%) KD of KL1 was confirmed, as described previously [31] (Figure 6Aa). Immunocytochemical staining for KL1 validated the effects we observed in mitotic spindles (Figure 6Ab). Significantly, the tau KD induced Chr-bridging in anaphase (Figure 7A) but no clear change was evident during metaphase. Anaphase Chr-bridging was found to be significantly inhibited by further expression of tau-KD resistant 2N4R. The mitotic index showed only a small increase in tau-KD cells (3.3 ± 0.4%, p<0.01), compared with the control cells (1.8 ± 0.2%).

We next focused on KL1-KD cells. During mitosis we found no clear aberration in Chrs (Figure 7A) as reported previously [31]. No significant increase in the mitotic index (2.0 ± 0.4%) was found either. Provocatively however, a double KD of tau and KL1 significantly repressed Chr-bridging (Figure 7A) compared with tau KD alone, suggesting that activated KL1 activity is causal for Chr bridge formation. One question arose regarding our observations of Chr-bridging in these experiments. Tau and a severing family protein are known to have functions not only via MT but also via their interactions with DNA [65, 66]. Hence, in addition to MT loss there is a possibility that Chr-bridging might also be caused by an alteration in DNA structures.

To examine whether excess KL1 activity actually causes disconnection of Chrs from mitotic spindles via a loss of Kt MTs, we adopted a previously described method [67] (Figure 7B). Briefly, HMECs were treated with monastrol and forced to produce monopolar spindles, in which the spindle to Chr connectivity is preserved. Cells are then arrested at prometaphase/metaphase, and are expected to encounter more severing events than normal mitosis. The rosette-like alignment of Chrs creates an open space outside of the spindles, in which detailed analyses of Kt-MT interactions in disconnected Chrs are feasible. In accordance with the original method [67], we classified the monopolar spindles into two categories, namely, “complete” and “incomplete”. We modified the definition of the term “incomplete” to indicate that the spindle has no obvious MT connection to the disconnected Chrs.

Most of the control cells showed a “complete” spindle with a rosette-like Chr alignment. ACA staining detected that most of the Kts were accompanied by MTs. Tau KD significantly induced “incomplete” spindles. In minor “incomplete” cases two types of disconnected Chrs coexisted; one with and one without a Kt fiber. Interestingly, the double KD of tau and KL1 reduced the formation of “incomplete” spindles (Figure 7B). These data indicate that under a tau KD, the excess KL1 activity leads to a loss of Kt fibers and results in a disconnection of Chrs from the mitotic spindle.

We further assessed micronucleation in HMECs. As shown in Figure 7C, whole Chr micronucleation in tau-KD cells was detectable, which was rescued by further expression of tau-KD resistant 2N4R or inhibited by KL1 KD. A KL1 KD alone showed no effect on micronucleation.

Aneuploidy can cause a p53-dependent G1 cell-cycle arrest [52], followed by apoptosis in human cells. To eliminate these effects, we knocked down p53 (Figure 6Aa). Neither a p53 KD nor double KD of KL1 and p53 caused any significant elevation in micronucleation. However, under a p53 and tau double KD we observed about a 2-fold increased level of micronucleation, which was inhibited by further KL1 KD (Figure 7C). These data support our hypothesis that in tau-KD cells, the excess MT severing by KL1 causes aneuploidy.

### Reduced physical rigidity in tau-KD mitotic spindles

To further explore the relevance of mitotic spindles to the Chr aberrations, we assessed the physical strength of their MT architecture. We predicted that if there is excess of severing, the MTs in prometa/metaphase spindles would suffer damage even under the operation of an SAC. Previous data on intermediate damage to spindle MTs by MT severing [25] support this idea. We applied a previously described method to examine the physical strength of purified mitotic spindles [68]. HMECs were synchronized at mitosis, collected, and the cytoskeletal architecture except for MTs, DNA, RNA, and membranes were removed by biochemical manipulation.

**Figure 8:**
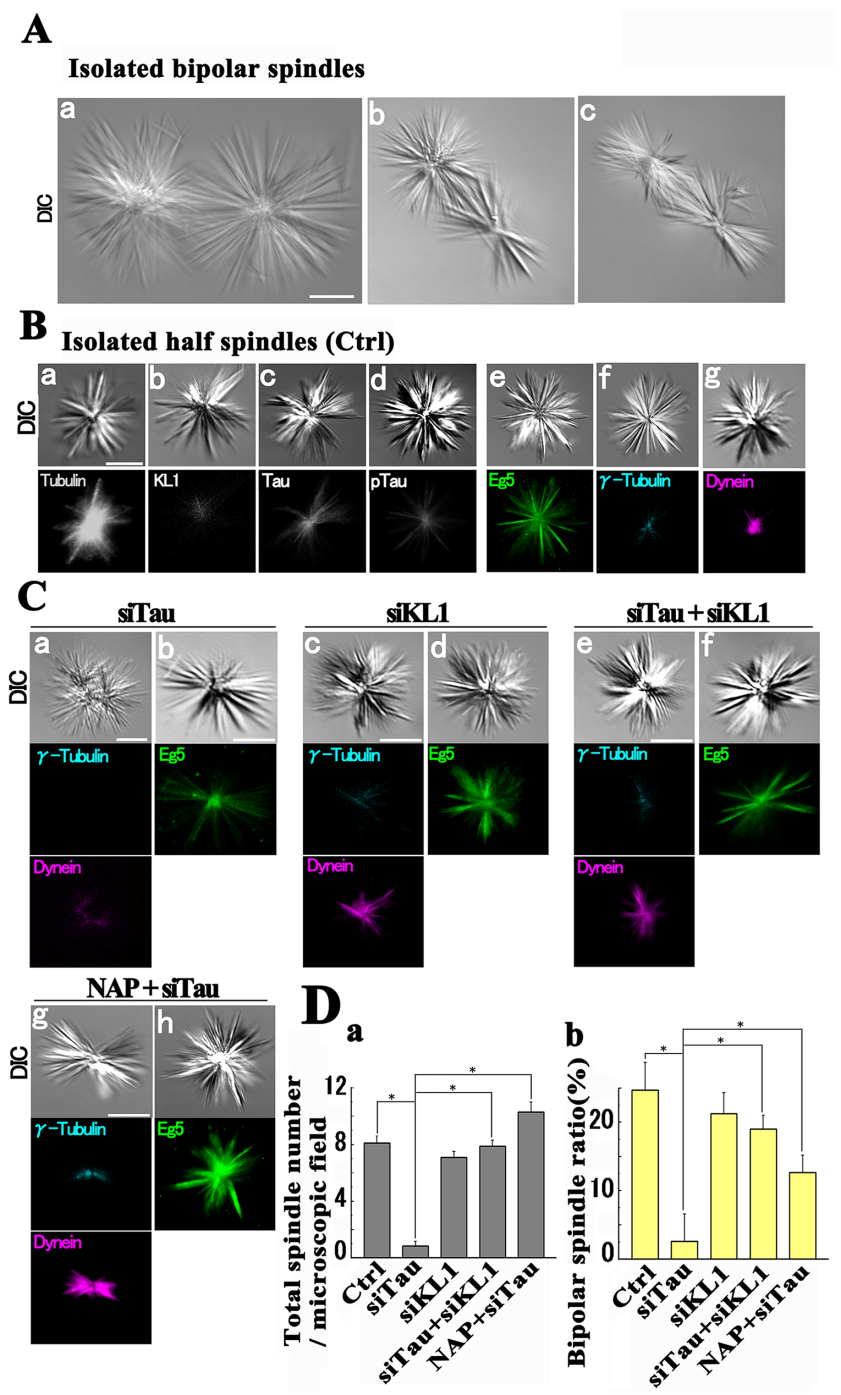
Isolated spindles under mechanical stress **A.** Representative difference interference contrast (DIC) images of isolated bipolar spindles (a-c). **B.** Immunostaining of half spindles. The tubulin (a) and tau (c) signals in the entire spindles and the KL1 (b) signals in the central region were detected. Phospho-tau signals (d) were also detected. Eg5 (e) showed two distinct patterns. The majority of these patterns showed distal tapering whilst the minority (static Eg5) showed a relatively distal enrichment. Gamma-tubulin signals showed a central spot (f). Dynein signals converged on the pole (g). No difference was observed between control siRNA transfected cells (A and B) and non-transfected cells. **C.** In roughly 50% of tau-KD spindles (siTau; a and b), the lack of MT convergence (a) was accompanied by the loss of γ-tubulin and dynein signals. In the other cases (b), static Eg5 signals were frequently lost. Under KL1 KD (siTau+siKL1) or NAP treatment (NAP+siTau), these aberrant phenotypes were mostly corrected. **D.** Quantification of the isolated spindles. (a) Total spindle numbers and (b) bipolar spindle percentages (*n*=3). Bars, 5 μm. The asterisks indicate significant differences (Student's *t*-test, *P*< 0.01).

High speed centrifugation (~1500 g) has been utilized previously to provide the cellular cytoskeleton with a mechanical stress [69, 70]. Isolated spindles are known to take two major forms, bipolar and half spindles. The former is relatively fragile for which too much mechanical agitation is known to cause more half spindle formation [71]. Consistently, we only detected half spindles when we centrifuged our spindle preparations at 3000 g. Therefore the isolated spindles were subjected to centrifugation at 1500 g for a prolonged time. We detected both bipolar and half spindles as shown in Figure 8 (about 25% and 75%, respectively). The bipolar spindles showed a line symmetry and comprised two half spindles connected by a middle region consisting of overlapped MTs (Figure 8A). Each half spindle showed a sphere like morphology (Figure 8Aa) and there were some compressed forms (Figure 8Ab and 8Ac). These morphologies were consistent with those obtained by a previous computer-based simulation that reflected the functions of MAPs [72].

We next performed immunostaining with a focus on the half spindles because they were observed irrespective of the experimental conditions. In the control siRNA-treated spindles, we detected strong tubulin signals in the whole spindle. The KL1 signals were observed to surround the center while both total tau and phospho-tau (153T) signals showed a broader distribution (Figure 8B). The Eg5 signals showed a centrally strong and distally weak pattern, and a relatively distally shifted pattern. The latter may correspond to ‘static Eg5’. The γ-tubulin signals showed a central convergence into a single dot. The dynein signals showed polar accumulation (Figure 8B). The γ-tubulin, dynein, and static Eg5 signals were used to show the centrosomal, polar, and relatively distal regions of the spindle, respectively.

Strikingly, in tau-KD cells we observed drastic reductions in the total number of spindles (87.5%) and in the percentage of bipolar spindles (99.7%; Figure 8C and 8D). Interestingly, about 50% of the spindles showed a loss of the γ-tubulin dot and weakened dynein signals that had also lost their central convergence. Consistently, the difference interference contrast images also detected ambiguous MT convergence. In the other cases, Eg5 staining in the tau-KD background detected a frequent loss of static-Eg5 (Figure 8C). These data suggested that both the distal and centrosomal parts of the spindle were missing. In contrast, the spindles from KL1-KD cells showed no such prominent changes (Figure 8C). Provocatively however, under a double KD of tau and KL1 (Figure 8C and 8D), we observed a normal structure when compared with the tau-KD cells. Both the total number and the percentage of bipolar spindles returned to the control levels in the double KD background. Also in these double KD cells, the dynein signals recovered their original intensity and polar location, the Eg5 signals recovered the “static” pattern seen in the control cells, and the γ-tubulin signals regained a centrally dotted pattern. These data suggest that tau-KD-induced spindle dysregulation under stress is caused by KL1.

### Karyotype analysis of tau-KD HMECs

**Figure 9:**
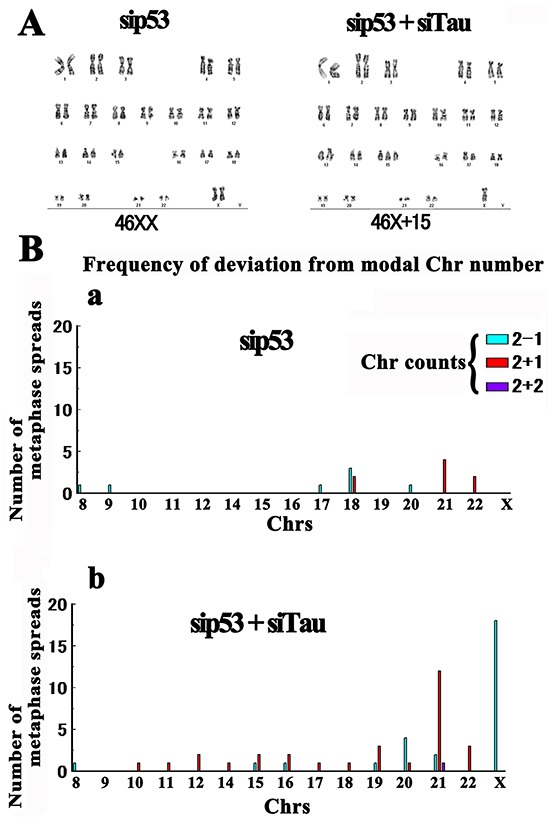
Karyotyping of tau-KD HMECs. Cells were treated with siRNAs and karyotyped using G banding **A.** Representative karyotypes. Under a p53 KD (sip53; 46XX), the majority (90%) of cells showed a normal karyotype (aneuploid level, 10%). Under a p53 and tau double KD, 25% of the diploid cells exhibited aneuploidy (sip53+siTau; 46X+15). **B.** Frequencies of deviation from the modal number for each Chr indicated by sky blue (loss of one Chr), red (gain of one Chr), and deep blue (gain of two Chrs) bars.

The micronucleation we observed in tau-KD HMECs was suggestive of aneuploidization. To assess this further, we performed karyotyping. HMECs were considered to be an appropriate culture system in this regard because they have a normal human karyotype [73]. First, we karyotyped 64 control siRNA-transfected cells. Of the 62 diploid cells observed, 56 (90%) were normal diploid (46XX) and 6 exhibited random gains or losses of 1 or 2 Chrs (10%). We next karyotyped 98 p53 KD cells. Of the 91 diploid cells observed, 82 (90%) showed a normal karyotype and 9 (10%) were aneuploid (Figure 9). This finding was consistent with that of a previous study showing that a p53 deficiency alone does not cause aneuploidy [74]. There was only a small Chr 21 triplication peak (4 cases out of 91; 4.3%) for these cells as was also observed in the controls (2 cases out of 62 diploids; 3.2%).

By contrast, double tau-/p53-KD cells exhibited increased aneuploidization. We karyotyped 136 metaphases of these cells and of the 128 diploid cells observed, 96 were normal and 32 exhibited aneuploidy (25%). Unexpectedly, we detected a marked peak (18 cells; 14%) indicating a loss of one X Chr. A second Chr 21 triplication peak was also detected (12 cells; 9%), consistent with a previous study [39] (Figure 9). Finally, although to a smaller degree, we observed a similar pattern of aneuploidization in single tau-KD cells (Supplementary Figure S6), reconfirming our micronucleation assay results.

### A strategy for diminishing CIN in tau-deficient HMECs

NAP (Asn-Ala-Pro-Val-Ser-Ile-Pro-Gln) is a membrane permeable peptide whose sequence is contained within the neurotrophic factor activity-dependent neuroprotective protein. NAP has been shown to protect neurons against a variety of insults and to bind MTs [75, 76] but have no effects on the proliferation of cells [77]. NAP has also been shown to be tolerable in clinical trials [78]. The beta-III tubulin isotype binds to NAP [75] and this association is needed for NAP to exert its effects. Previously, we observed that NAP inhibited MT severing in the absence of tau [44]. Thus, we predicted that NAP could be used in HMECs.

We first performed the monopolar preparation to test the effects of 1, 30, and 1000 nM of NAP on the loss of Kt MTs and found that the 30 nM dose gave the maximal effect (55% inhibition). However, NAP alone caused neither a change in the mitotic index nor spindle aberrations (Figure 7B). We then isolated the mitotic spindles to further evaluate the effects of NAP. Strikingly, compared with the control siRNA, we observed a 1.3-fold increase in the total number of spindles in NAP-treated tau-KD cells and also that NAP had clear effects in preserving bipolarity (Figure 8D). The regional markers we tested also recaptured their original distributions upon NAP exposure (Figure 8C). In the same settings, we observed a significant (62%) reduction in anaphase Chr bridges under NAP treatment (Figure 7A) and a pronounced suppression of micronucleation (83% inhibition) even under a p53 KD (Figure 7C). These data indicate that NAP may be applicable to strategies designed to prevent transformation driven by tau deficiency-induced CIN.

## DISCUSSION

In our current study, we evaluated the relationships between tau and KL1 in early breast carcinogenesis. In the KL1(L123V) mutant, the mutated residue lies within the N-terminal half of the KL1 protein. The N-terminus has been considered to control MT binding [32, 79], so one possibility is that this mutation may alter the affinity of KL1 to MTs. However, we did not observe any changes in the intracellular distribution of GFP-KL1(L123V), compared with GFP-KL1wt in our current experiments.

Our current hypothesis is that the leucine residue may be important for interaction with possible Kt-MT specific tubulin post-translational modifications [80] and is currently under investigation. Among the known CIN genes, the Kt proteins Hec1 and CENP-E are known to have affinities for MTs. Hec1 overexpression and a CENP-E haploinsufficiency in genetically engineered mice have been demonstrated to cause late onset tumorigenesis through CIN [36, 49, 50]. The MEFs derived from these animals exhibited a Kt-MT reduction [81] and triad characteristics [36, 49–51]. These characteristics were interpreted as tumor promoting CIN. Hence, the triads in our current experiments can be said to be predictive of CIN and are consistent with the transformation and micronucleation events that we observed.

With regard to the roles of tau in mitotic spindles, we focused on Kt fibers because of their essential role in Chr segregation. The MTs connecting the Kts to the poles are known to be arranged in discrete bundles [61–63, 82] and show hexagonal packing on an electron micrograph [63, 83]. The existence of physically linking “cross bridges” has been reported within or near these bundles [83]. These cross bridges are thought to be involved in lateral interactions which structurally link individual MTs into a mechanically coherent bundle that can better transmit force to the Chr. Notably, these coherent effects have been demonstrated in living grasshopper spermatocytes using a micromanipulation needle [84].

In the mitotic spindle, due to its intrinsic dynamic nature, lateral interactions are considered to be transient [82]. Continuous but random transient lateral interactions between MTs are thought to produce the mechanical cohesiveness of the Kt fibers/mitotic spindles. In our current experiments, we detected tau on the Kt fibers and also observed that a tau KD disrupted these fibers under cold treatment which will inhibit the ATPase activity of motor proteins. Taken together, we speculate from these results that tau may contribute to the mechanical strength of Kt fibers.

In the mitotic spindles, we detected the KL1-induced loss of Kt MTs under drug treatment. We also detected a decrease in the physical strength of isolated mitotic spindles. Of relevance to these results would be that on the MT lattice, the initial attack of nematode katanin is known to make incomplete scissions the number of which is more than 10 times higher than complete ones and they have been observed throughout the spindle [25]. In our current study, both centrosomal and distal markers of the spindle lost their original localizations under a tau KD. Therefore, we speculate that the loss of tau causes intermediate damage throughout the spindle.

The potentially MT destructive spindle environments shown by our current experiments strongly support the relevance of the mitotic spindle to the observed Chr-bridging in anaphase. The number of Kt MTs is known to decrease with the progression of anaphase [62, 85]. Therefore, besides the lack of an SAC, another factor in anaphase may cause excess MT severing (Supplementary Figure S7).

We observed significant aneuploidization in tau-KD HMECs. The karyotypes in these cells were also marked by Chr 21 triplication and loss of one Chr X. Normally, one of the two X Chrs in a human female cell is inactivated (Xi). There are reports of a frequent loss of Xi in human breast cancer cells [86] and a proposed causal role for the loss of Xi in a distinct subtype of human breast carcinoma with a poorer prognosis [87]. Therefore there is a possibility that unbalanced KL1/tau-induced loss of one Chr X contributes to breast carcinoma pathogenesis. Two possibilities are suggested by previous studies as to why there is selective loss of an X Chr. First, the Xi may have inhibited Kt functions in terms of MT connection or nucleation and may reduce the number of Kt MTs [88]. Second, as reported previously, the sex Chrs may have a tendency to have smaller number of Kt MTs than autosomes during anaphase [89].

Our suggested strategy to prevent tumor progression using NAP may be relatively distinctive. The pharmacological mechanisms of recent anti-tumor drugs are based on cytotoxicity. By contrast, our present strategy would allow potential tumor ancestor cells to persist. We believe that creating a symbiosis with non-hazardous tumor cells can be realized only when their evolutionary power is nullified. Potentially, our current findings could have more universal significance. The salivary tissues, which also express tau, may deserve some attention in that both mammary and salivary tumors that develop in *ras*/*p53*^-/-^ mice commonly showed extensive genomic instability [90]. The loss of *APC* gene function is known to be causal for colon carcinogenesis. A recent neuroscience study revealed that APC is also required for neuronal migration during brain development [91]. Without APC, tau loses its regulatory effects on katanin. The tau and katanin relationships in the developmental brain could have relevance to colon carcinogenesis.

## MATERIALS AND METHODS

### Antibodies and reagents

Rabbit polyclonal antibodies against KATNAL1 (KL1) (HPA046205, Sigma, St. Louis, MO, USA), dynein (DYNC1LI1) (GTX120114, GeneTex, Irvine, CA), Eg5 (KIF11) (GTX109054, GeneTex), Neuron specific β-Tubulin III (GenScript, Piscataway, NJ), H-Ras (GeneTex), ErbB2 (GeneTex), GFP (Sigma), Flag (Cell Signaling Technology, Danvers, MA) were used. Rabbit polyclonal antibody against phospho-Tau (T470) (sc-16898, Santa Cruz, Santa Cruz, CA) was used as anti-phospho-Tau (153T: amino acid number in 2N4R (441 amino acids)) antibody. Rabbit monoclonal anti-p53 antibody (clone 7F5, Cell Signaling Technology) was used. Goat polyclonal antibody against Tau (GTX11107, GeneTex) was used. Mouse monoclonal antibodies against KL1 (clone 1A7, OriGene Technologies, Rockville, MD), α-Tubulin (DM1A, NeoMarkers, Fremont, CA), Cy3-conjugated-β-Tubulin (Sigma) (used for general tubulin staining), γ-Tubulin (Sigma), β-Actin (Sigma), and Flag (TransGenic Inc., Kobe, Japan) antibodies were used. Two mouse monoclonal phospho-independent anti-Tau antibodies (T1029, USBiological, Swampscott, MA) and (Tau-13, Santa Cruz) were used for total tau detection. Human anti-centromere antibody (Antibodies Inc., Davis, CA) was used. DAPI (4,6-diamidino-2-phenylindole dihydrochloride hydrate) (Sigma), Nocodazole (Sigma), Paclitaxel (Wako, Osaka, Japan), Colcemid (Life Technologies, Carlsbad, CA), MG132 (Cell Signaling Technology), and Monastrol (Cayman Chemical, Ann Arbor, MI) were used. NAP peptide (Biorbit, San Francisco, CA) was aliquoted and stocked as a 1 mM solution in 75% dimethyl sulfoxide at -20C.

### Expression constructs

Human KL1 expression vector was purchased (RC220944, OriGene) and the KL1 protein was expressed under pEGFP-C1 (Clontech, Mountain View, CA, USA) (GFP-KL1) and pMXs backgrounds. The breast cancer derived mutation KL1 (L123V) [48] and loss of severing mutation (L288V) [33] were introduced by GeneArt Site-Directed Mutagenesis PLUS kit (Life Technologies). The expression vectors of rat katanin (pEGFP-C1-p60 katanin) and mouse spastin (pEGFP-C1-M85) were kind gift from Dr. PW. Baas, Drexel University, Philadelphia, PA. Human tau expression vectors, pRC/CMV-Flaghtau441wt (2N4R), pRC/CMV-Flaghtau352wt (0N3R), pRC/CMV-FlagPHPtau441 (PHP), pHSV-GFP-htau352, and pHSV-GFP-htau441wt (GFP-Tau) were kind gift from Dr. R. Brandt, University of Osnabrück, Barbarastrasse, Germany [92-94]. The major eight mitotically phosphorylated amino acids in tau (T153, T181, S202, T205, T212, S214, T217, and T235) [12] were substituted with glutamates by the site-directed mutagenesis kit: pRC/CMV-FlagMHPtau441 (MHP). Tau-siRNA resistant mutant tau (Tau rst. mt) was constructed by introduction of three nucleotide changes (neutral mutations) into the siRNA-targeted sequence in pRC/CMV-Flag-htau441 by the site-directed mutagenesis kit using the primers (Forward: atccctaggcaacatccatc, Reverse: gggatccacacttggaggtc).

Authentic oncogene expression vectors ErbB2 (V659E) (: ErbB2mt) in pQCXIN and H-Ras (G12V) (: Rasmt) in pMXs were kind gift from Dr. K. Semba, Waseda University, Tokyo, Japan and Dr. T. Yamamoto, Okinawa Institute of Science and Technology Graduate University, Okinawa, Japan [47]. Both oncoproteins were expressed from pMXs vectors. For details, see Supplemental Methods.

### Cell culture, transfection and drug treatments

*RFL-6, cells*. Rat RFL-6 fibroblasts were purchased (Health Protection Agency, Porton Down Salisbury, UK), cultured as described previously [43, 44], and transfected with expression plasmids using the electroporation device NEPA GENE CUY21Pro-Vitro (Ichikawa, Chiba, Japan) with the setting of program as (Pp; 275V, 5 ms, 50ms, and Pd; 20 V, 50 ms, 50ms, 10 cycles). The transfection efficiency was about 30-40%. Other conditions about electroporation, plating, and culture of cells were as described previously [43, 44].

For transformation assay using RFL6 and Rat-1 cells (RIKEN, Ibaraki, Japan) (Rat-1 was also used for the major part of experiments), basically similar method was utilized as described previously [29]. We used drug (puromycin (Takara Bio, Shiga, Japan) and geneticin (Life Technologies)) selection in focus formation assay to increase total amount of the exogenous proteins. The transfection efficiencies were similar through focus formation experiments. Foci that appeared on monolayer 3–4 weeks after the initiation of the selection were counted. In the colony formation assay we plated 200000 cells in a soft agar in 6cm dish as described previously [95] and after 1.5 months colonies were counted under a phase contrast microscope. At least two independent clones were analyzed for each condition.

*Human mammary epithelial cells.* Human (female) mammary epithelial cells (HMEC, CC-2551, Lonza, Basel, Switzerland) (passage 7, PD 18, Lonza) were purchased. Cells were cultured in the medium supplied by the MEGM Bullet Kit (CC-3150, Lonza) at 37°C and 5% CO_2_ and according to the manufacture's instruction.

Transfection of HMECs with TN1.5M or Tau rst. mt expression plasmids and/or with siRNAs was performed using TransIT-X2 Dynamic Delivery System (Mirus, Madison, WI). For tau constructs expression cells were plated on 35 mm culture dish and 2.5 μg of the plasmid was transfected. The transfection efficiency was about 20-30%. For KD of KL1, Dharmacon siRNA smart pool (GE healthcare, Pittsburgh, PA) was used and a non-targeting control siRNA was used as a control according to the previous study [31]. For the KD of tau and p53, previously reported siRNAs [96] and [97] were used, respectively. The final concentrations of siRNAs were 35, 50, and 10 nM for KL1, tau, and p53, respectively. The fluorescent dye conjugated siRNA transfection showed nearly 100% transfection efficiency. For synchronization of HMECs, cells were treated with 0.13 μM nocodazole or 20 μM MG132 for 18 hours.

The monopolar preparation by monastrol treatment was performed as described [67]. After two days culture under the KD conditions cells were treated with 100 μM monastrol for 5 hours and then fixed. For NAP treatment, cells were transfected with siRNAs and treated with NAP simultaneously. Similarly, Chr-bridging detection and micronuclei detection were performed after two days culture. In karyotyping, based on the previous study [73], we restricted our analysis to cells at passages 10 and 11. After three days culture, HMECs were treated with 0.04 μg/ml colcemid for three hours, harvested according to standard cytogenetic methods as described [98]. GenASIs BandView system with the BandView software (Applied Spectral Imaging, Carlsbad, CA) was used for metaphase spreads capture and Chr analysis. In the control, 2 of 64 cells showed tetraploid. In p53 KD, 6 out of 98 cells showed tetraploid, while in tau+p53 double KD, 7 of 136 cells were tetraploid.

### Western blotting, immunoprecipitation, and immunoblotting of breast cancer tumor /normal tissue lysate test strip arrays

Western blotting was performed as described previously [29]. Immunoprecipitation was performed as previously described [29] with some modifications. To allow electrostatic interaction between projection domains [2] and to avoid S-S bonding in GFP-tau, we used low ionic strength lysis buffer with mild reducing reagent (NaCl: 10 mM, DTT: 1 mM) and to suppress non-specific interaction between proteins we washed beads 10 times with the lysis buffer. We also added 1 mM Na3VO4 to the lysis buffer.

Human Breast tissue lysate collections (ST2-6X-1 and 2; Proteus Biosciences, Ramona, CA) were purchased. The strips were immunoblotted with anti-KL1 and anti-total tau antibodies according to the manufacturer's instruction using ECL reagents and developed in X ray films. Totally 16 μg protein is loaded for each spot. The equal protein loads were confirmed by actin immunoblots. We restricted our analysis on primary ductal carcinoma with stages ranging from IIA to IV. Cases 1 and 2 correspond to T2-005-T/N-1 and T2-034-T/N-1 in Extract IDs of ST2-6X-1, respectively. Cases 3 to 7 correspond to T2-017-T/N-1, T2-018-T/N-1, T2-029-T/N-1, T2-046-T/N-1, and T2-048-T/N-1 in Extract IDs of ST2-6X-2, respectively. Three independent experiments were performed for each blot. The expression levels were evaluated by densitometry with the cut off values 1.5 and 0.67. The absorption of antibodies by antigens was performed as previously described [29] using GST-KL1 and GST-tau proteins expressed and purified in bacterial cells.

In the experiments to assess the molar ratio of tau to tubulin heterodimer on spindle of HMECs, we performed fractionation experiments by applying the previously established method with some modifications (Polymerized vs. soluble tubulin separation assay) [58] to mitotic cells. We synchronized HMECs with treatment of MG132 and collected mitotic cells by mitotic shake off. After wash, cells were lysed at 37 C for 20 minutes with MT-buffer [58] with 1.0% Triton X100. The tau and tubulin proteins contained in the polymerized (P) fraction were quantitatively analyzed. We used GST-tau derived protein and GST-tubulin protein expressed and purified in bacterial cells as standard proteins.

The protein amounts were quantified by densitometry using NIH ImageJ software.

### Immunofluorescence techniques

The immunostaining experiments were performed as described previously [43, 44]. For immunostaining of endogenous proteins in HMECs, ice cold methanol fixation was performed for KL1, γ-tubulin, dynein, and Eg5. For centromere staining with ACA, we fixed HMECs with ice cold methanol+acetone (1:1) for 15 minutes. For staining of total tau or phosphorylated-tau and general tubulin, cells were fixed and extracted with 2% paraformaldehyde, 0.1% glutaraldehyde, 0.2% triton X100 in PHEM [43] for 30 minutes at room temperature. Fluorescence signals were detected using a confocal laser scanning microscope, LSM 700 (Carl Zeiss, Thornwood, NY, USA) using Plan-Apochromat 40 or 63 x (oil) objective lenses with 1.3 or 1.4 apertures, respectively. The original magnifications were 400 or 630. To quantify MT levels, cells were simultaneously fixed and extracted to remove free tubulin and then immunostained as described previously [43]. Images to be compared were taken at identical settings of exposure time, brightness and contrast and analyzed with ZEN 2012 software. In MT sensitivity test, we chose medium expressers as described previously [43]. Measures of MT levels were taken as total fluorescence intensity per cell using the analytical command of intensity mean value in ZEN software. A newly introduced index total bundle levels were calculated as signal summation of bundle(s) in a cell divided by the area of the cell. MT bundles from all subcellular regions including the perinuclear region were analyzed. Statistics were done using Student's t-test. Values were expressed as arbitrary fluorescent units (AFUs). In monopolar preparation in HMECs, the disconnected chromosome is defined as that they are clearly detected outside of the spindles with a distance of more than one chromosome width from the closest chromosomes and are devoid of detectable Kt fibers. About 10% of monopolar spindles among whole cell population were detected for all experimental conditions. Irrespective of conditions there were minor skewed monopolar spindles.

### Mitotic spindle isolation under centrifugal stress

Purification of mitotic spindles from HMECs was performed using the established method [68] with some modifications. We synchronized HMECs with MG132 treatment. Before isolation the MG132 treated mitotically arrested cells mostly showed bipolar spindles (meta/prometaphase) with a slight increase in multipolarization in the immunostaining study. There were no observable differences in the efficiencies of synchronization among the various conditions. The collected mitotic cells (roughly 50000 cells) by mitotic shake off were immediately treated with 10 μg/ml paclitaxel for three minutes at 37 C. The isolated spindles were provided with mechanical stress by high speed centrihugation (1500 g for 15 minutes). Finally totally 20 μl of purified spindles were obtained in low-ionic strength buffer (1 mM Pipes. pH6.9, 10 μg/ml paclitaxel, 0.5% Triton X100: LIS). Four micro litters of the spindle were spotted per well on glass coverslips mounted in the bottom of 35-mm-diameter Petri dishes with holes drilled in the bottom. The spindles were immediately and gently mixed with 70 μl of liquid state 2% low melting temperature agarose (Takara Bio) (in LIS without Triton X100) held at 37 C on the glass coverslips and then put under 4 C for 30 minutes to solidify the gel. Then spindles embedded in the gel were fixed with the fixative (2% paraformaldehyde, 0.1% glutaraldehyde in PHEM) for 30 minutes at room temperature. For immunofluorescent analysis, for some antigens, we further added fixation by ice cold 70% methanol for 15 minutes. Immunostaining of purified spindles were performed basically in the same manner as the standard immunostaining experiments with extension of time period for each steps to attain efficient diffusion of antibodies through the gel (incubation time with the antibodies: overnight (at room temperature); wash time with PBS: two hours for each wash). The stained spindles were analyzed under laser confocal microscope as written in immunofluorescence techniques. The spindle number was counted under low magnification microscopic field. Randomly selected four fields were analyzed.

### On line data acquisition

We used 2012 version data set of breast cancer in an online database [45]. For the examination of prognostic properties of tau we analyzed totally 993 patients who were not systemically treated. The results regarding the probability of recurrence free survival (RFS) showed better prognosis in the subpopulation with high tau expression (n=581) than low expression (n=412) with hazard ratio of 0.73 (95% CI= 0.59-0.91, logrank P = 0.0043). Then the analyzed patients were restricted to those being ERα positive, treated with endocrine therapy but without chemotherapy (Figure 1A). Among totally 690 patients who fulfilled the restrictions, the patient group with high tau expression (n=496) showed significantly higher RFS probability compared with low expression (n=194) (HR=0.56 (95% CI=041-0.77), logrank P=0.00025). In the further analysis, the subdivision of primarily ERα positive patients depending on the ERα expression levels again resulted in no significant difference (HR=1.14 (95% CI=0.84-1.54), logrank P = 0.39).

## SUPPLEMENTARY MATERIALS FIGURES



## Abbreviations

Kt: kinetochore
MT: microtubule
Chr: chromosome
MAP: microtubule associated protein
KL1: katanin-like1
CIN: chromosome instability
SAC: spindle assembly checkpoint
