# Supplementary material for: The mitotic tensegrity guardian tau protects mammary epithelia from katanin-like1-induced aneuploidy
Source: Oncotarget. 2016 Jul 20;7(33):53712–34. doi: 10.18632/oncotarget.10728 (PMC5288216; doi:10.18632/oncotarget.10728)
Supplement: Supplementary file 1 [file oncotarget-07-53712-s001.pdf]

The mitotic tensegrity guardian tau protects mammary epithelia from katanin-like1-induced aneuploidy

SUPPLEMENTARY METHODS

Expression constructs (details)

Flag-tagged C-terminus of tau containing MTB domain (amino acids 245-441 in the number of 2N4R) expression vector pRC/CMV-Flaghtau C-terminus (TC) was constructed by insertion of hemi-blunted PstI-NheI fragment from pRC/CMV-Flaghtau441 to hemi-blunted Bpu1102I-NheI cut pRC/CMV-Flaghtau441vector. The same fragment with N-terminal GFP-tagged version (pHSV-GFP-htau C-terminus (GFP-TC)) was constructed by insertion of the fragment into hemi-blunted BglII-NheI-cut pHSV-GFP-htau352. pRC/CMV-Flag tau modified N-terminus (TN1.5M: Flag-tagged tau 1-196 fragment with amino acids substitutions T153 and T181 to Es) was generated by PCR mediated spontaneous deletion of 1512th cytosine (nucleotide number in pRC/CMV-FlagMHPtau441), resulting in the intermediate stop codon. A big tau cDNA (open reading frame composed of exons 2, 4, extended 4A [1], 5-7, and 9-13) was synthesized from human skeletal muscle total RNA (Clontech) by RT-PCR using primers for exon 2 and exon 13 sequences of the human tau gene (MAPT) (forward: gctgagccccgccaggagttcg, reverse:gctagcgtggcgagctggggc), subcloned into the pCR-Blunt II-TOPO vector (Clontech), then sequenced. pRC/CMV-Flaghbigtau (Big) was constructed by the insertion of Bpu1102I-NheI fragment of big-tau in TOPO vector into pRC/CMV-Flag-htau441 cut by the same set of restriction enzymes. Bacterial expression and purification of GST-tau (3-441) and GST-KL1 were from pGEX4T-1 while GST-α-tubulin was from pGEX4T-2 background. GST-tau derived protein was used as the standard. The structures of the constructs were confirmed by sequencing.

(Constructs used in Figure S5): pRC/CMV-Flag tau N-terminus (TN1) was constructed by self-ligation of blunted SacII-NheI-cut pRC/CMV-Flaghtau441. pRC/CMV-Flag tau modified N-terminus (TN1M) was constructed by self-ligation of blunted SacII-NheI-cut TN1.5M. pRC/CMV-Flag tau N-terminus with the deletion (TN1Δ: amino acids 4-17-deleted TN1) was constructed by self-ligation of blunted Bpu1102I-BsiWi-cut TN1. pRC/CMV-Flag tau N-terminus (TN1.5) was constructed by deletion of 1512th cytosine in pRC/CMV-Flaghtau441, resulting in the intermediate stop codon by site-directed mutagenesis. pRC/CMV-Flag tau N-terminus (TN2) was constructed by self-ligation of blunted BstXI-NheI-cut pRC/CMV-Flaghtau441.

REFERENCE

1. Wang J, Tse SW, Andreadis A. Tau exon 6 is regulated by an intricate interplay of trans factors and cis elements, including multiple branch points. J Neurochem. 2007; 100: 437-445.

SUPPLEMENTARY FIGURES

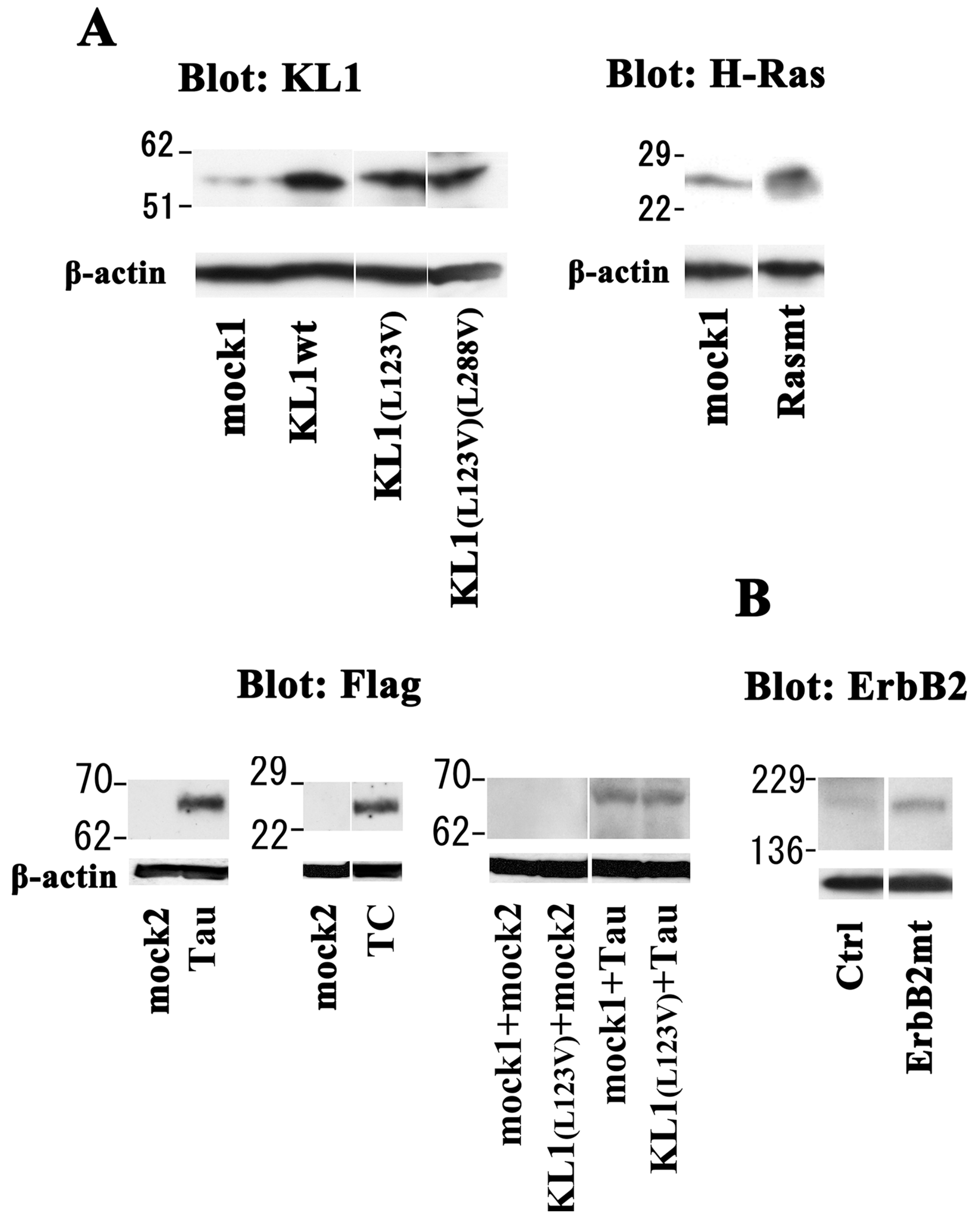

Supplementary Figure S1: Exogenous protein expression analysis in the transformation studies. A. The stably KL1wt, KL1(L123V), and KL1(L123V)(L288V) expressing RFL6 cells were analyzed for their KL1 variants protein expressions by western blots with anti-KL1 antibody (KL1). The stably Rasmt expressing RFL6 cells were analyzed for their ras protein expression by western blots with anti-H-Ras antibody (H-Ras). The stably tau (2N4R: Tau) and TC expressing cells were analyzed for their tau protein expressions by western blots with anti-Flag antibody (Flag). The stable KL1(L123V) expresser was further transfected with 2N4R expression plasmids and stably KL1(L123V) and 2N4R co-expressing cells were selected under geneticin treatment. The selected clone was analyzed for their tau protein expression by western blots with anti-Flag antibody (Flag). **B.** Transiently ErbB2mt transfected RFL6 cells were analyzed for their ErbB2 protein expression by western blots with anti-ErbB2 antibody (ErbB2). β-actin was used as a loading control.

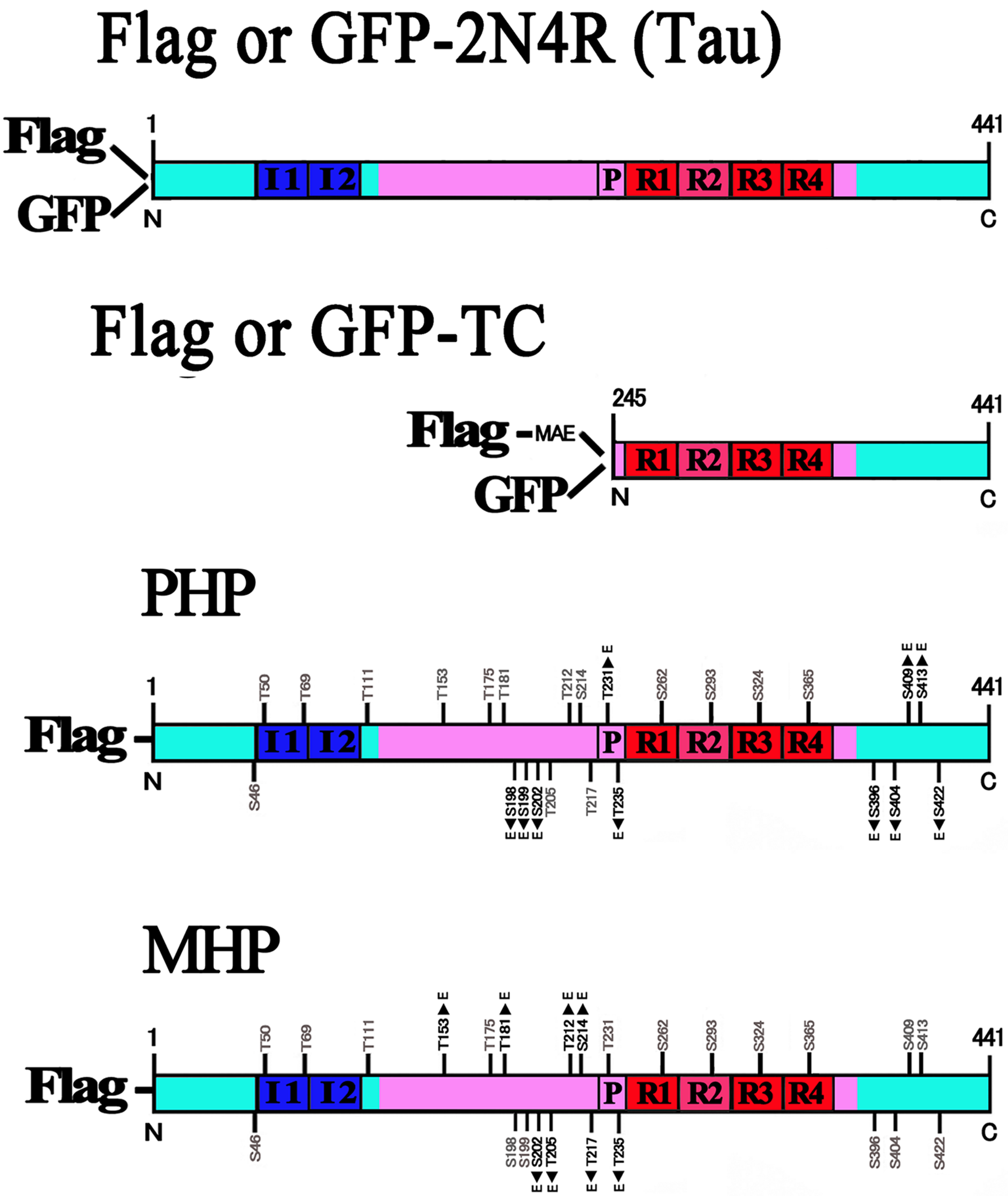

Supplementary Figure S2: Structures of TC and MHP. Human 2N4R tau (total length: 441 amino acids length) derived constructs. 2N4R tau is Flag- or GFP-tagged (2N4R (Tau) or GFP-2N4R (-Tau)). The C-terminal region of tau which consists of 245-441 amino acids of 2N4R is Flag- or GFP-tagged (TC or GFP-TC). PHP includes ten serine or threonine residues (S198, S199, S202, T231, T235, S396, S404, S409, S413, S422) substituted with glutamates while MHP includes eight serine or threonine residues (T153, T181, S202, T205, T212, S214, T217, T235) substituted with glutamates.

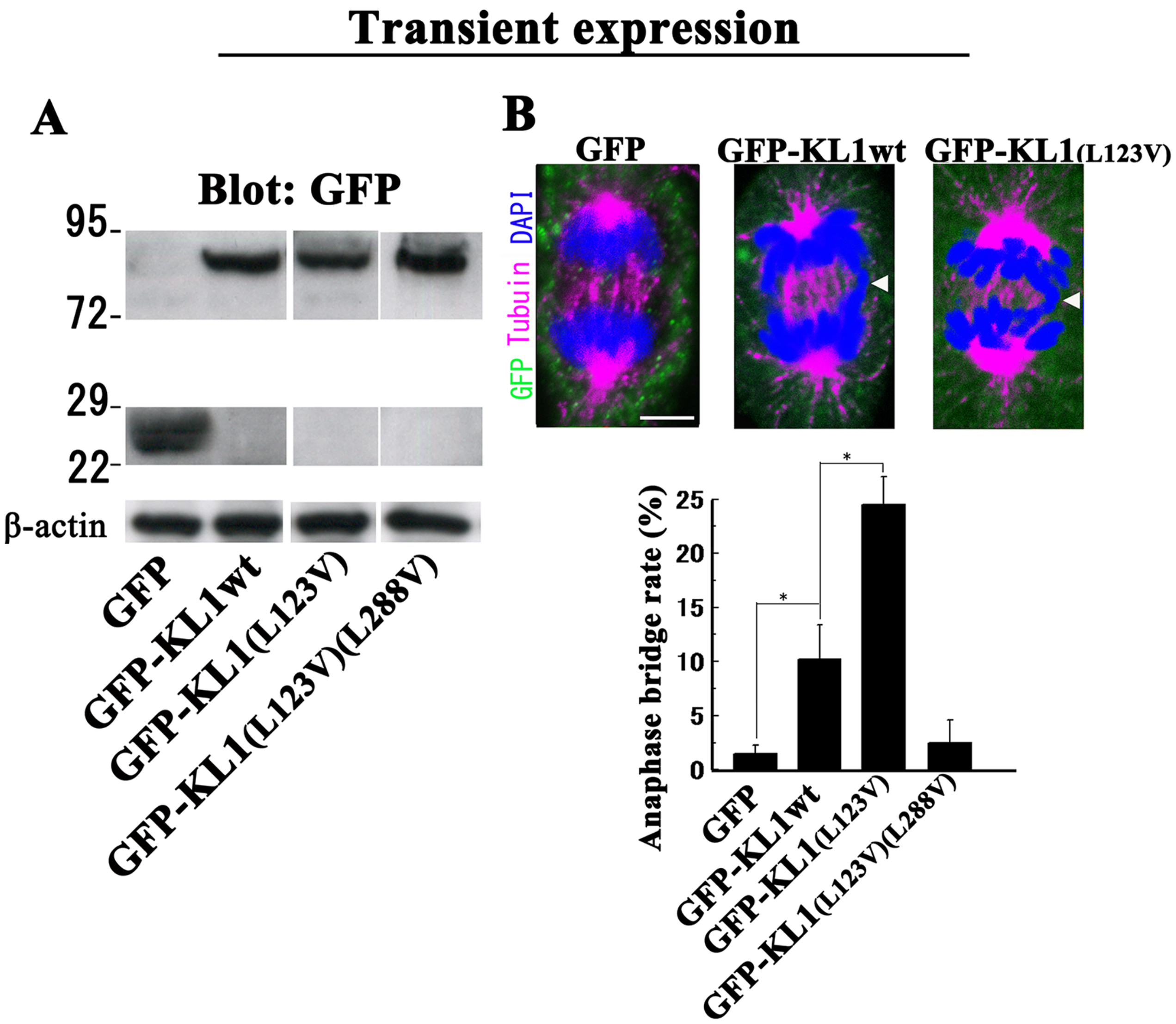

Supplementary Figure S3: Effects of transient expression of KL1wt and KL1(L123V) on RFL6 cells. A. RFL6 cells were transiently transfected with plasmids for expression of GFP-tagged KL1-derived constructs (GFP-KL1wt, GFP-KL1(L123V), and GFP-KL1(L123V)(L288V)). The whole cell lysates were subjected to western blots with anti-GFP antibody. β-actin was used as a loading control. **B.** RFL6 cells were transiently expressed with GFP-KL1wt, GFP-KL1(L123V), and GFP-KL1(L123V)(L288V). Stainning for tubulin and DNA detected significant anaphase Chr-bridging (arrowheads) in GFP-KL1wt and GFP-KL1(L123V)(L288V) expressing cells compared with the control GFP expressing cells (GFP). The severing null double mutant GFP-KL1 (L123V)(L288V) showed no significant increase in Chr-bridging. Bar, 5 µm. The graph shows quantification of anaphase Chr-bridging (>40 cells were counted, *n*=3). The asterisks indicate significant differences (Student's *t*-test, *P*< 0.01).

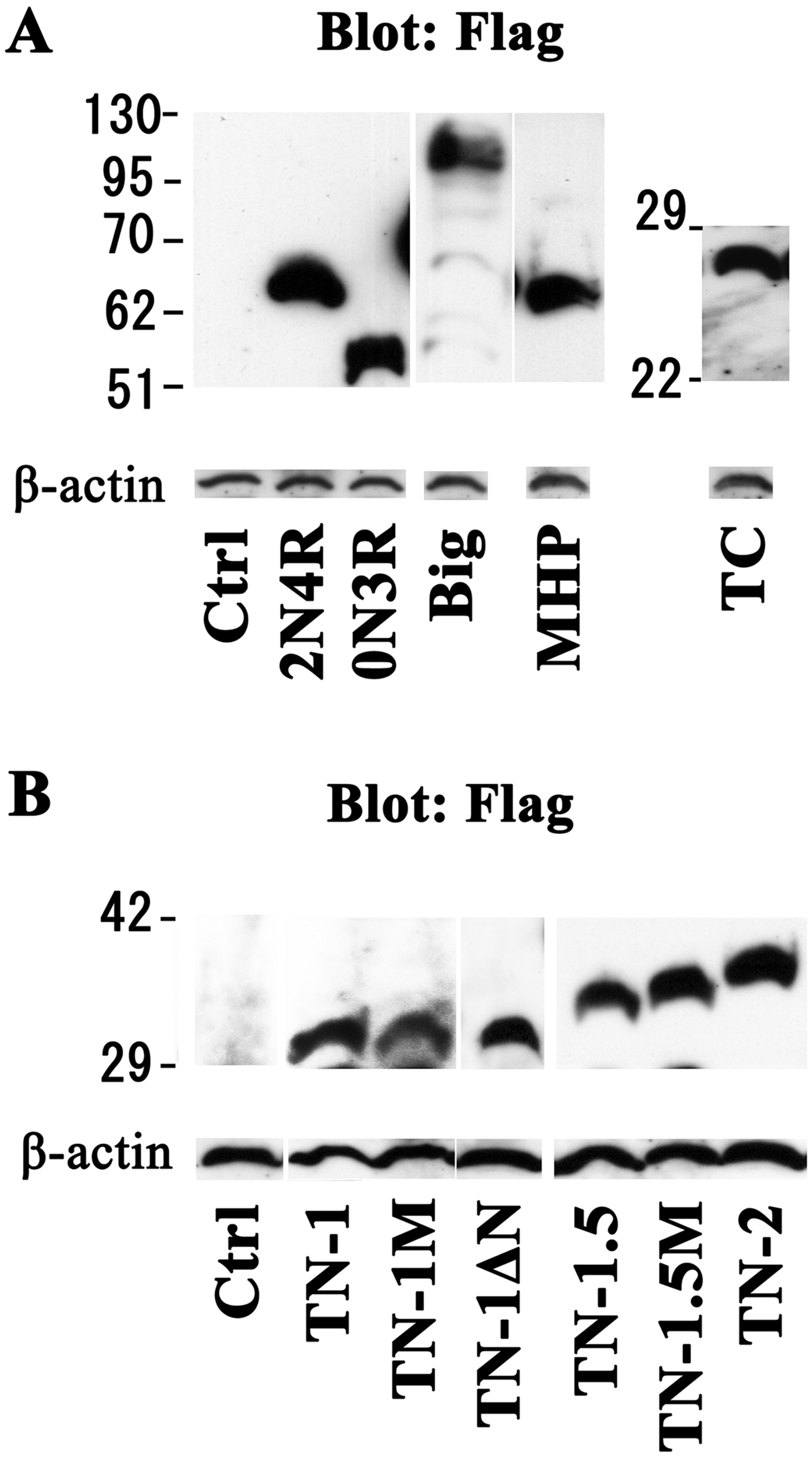

Supplementary Figure S4: Exogenous proteins expression analysis in MT sensitivity tests. A. Tau constructs protein expressions in RFL6-based MT sensitivity tests. RFL6 cells were transiently transfected with the indicated tau constructs and the whole cell lysates were subjected to western blots with anti-Flag antibody. **B.** Tau N-terminus constructs expressed in RFL6 cells. Cells were transiently transfected with six distinct tau N-terminus constructs used in Figure S5 and the whole cell lysates were subjected to western blots with anti-Flag antibody. β-actin was used as a loading control.

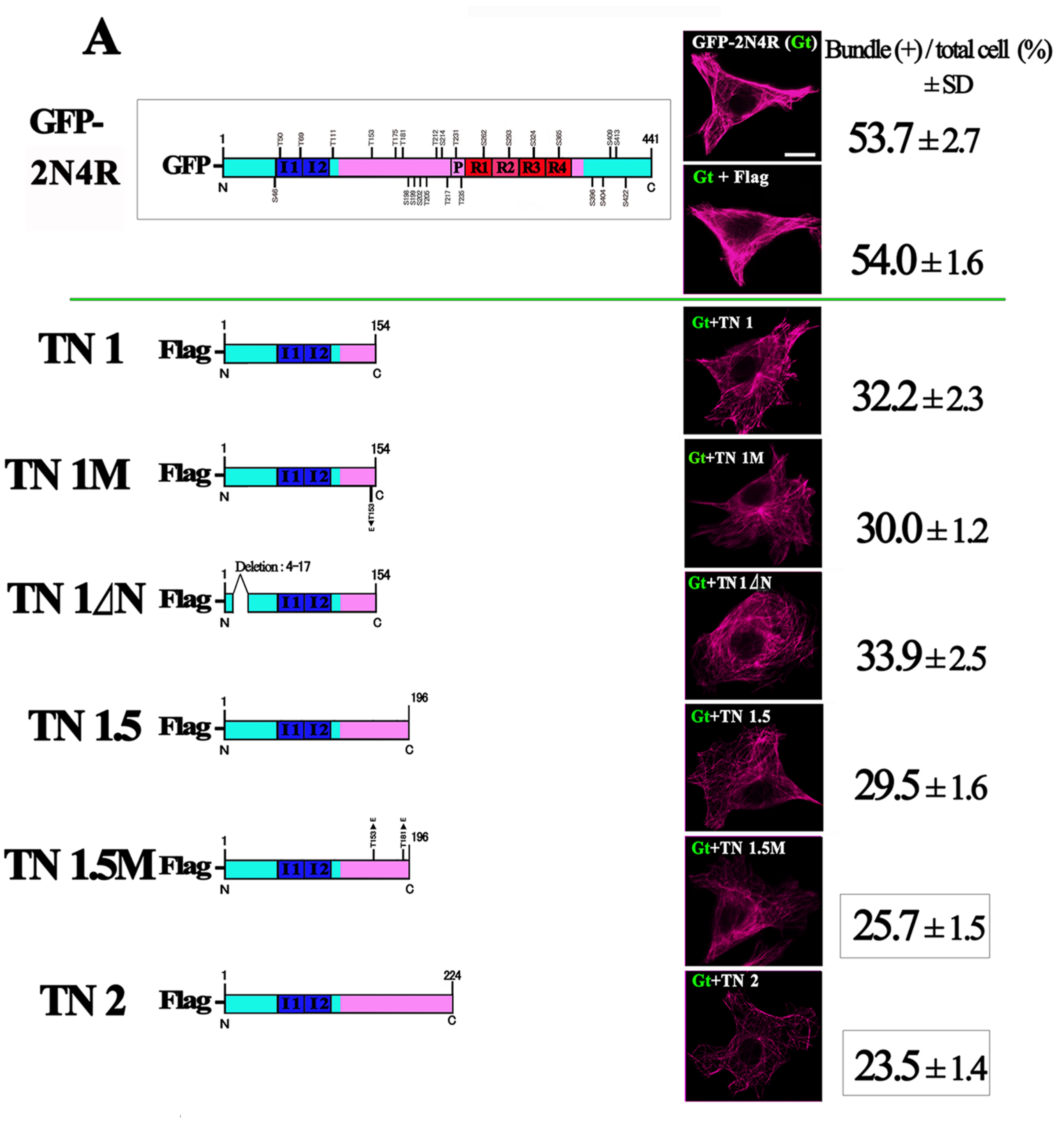

Supplementary Figure S5: Selection of the MT bundling inhibitor. A. The selection of the tau N-terminus construct with the efficient inhibitory effect on GFP-2N4R induced MT bundling in RFL6 cells. GFP-2N4R (Gt in the images) expression or GFP-2N4R+Flag co-expression significantly induced similar MT bundling levels with more than fifty percents cells exhibiting at least one bundle compared with 12.0 ± 0.3% in the control cells (P< 0.01). Co-expression of GFP-2N4R and one of N-terminus constructs significantly reduced the percentages of cells possessing MT bundle(s). Upper two images showing general tubulin staining (magenta): control cells expressing GFP-2N4R or GFP-2N4R+Flag. The third to eighth images: cells co-expressing GFP-2N4R and the indicated tau N-terminus construct. The rightmost figures show the percentage ± S.D. of cells with MT-bundle(s) for each condition. (40<cells were analyzed, n=3). Compared with the control (GFP-2N4R+Flag), all combinations of GFP-2N4R+N-terminus co-expressing cells showed significant inhibition (Student's t-test, P< 0.01). Among the six constructs, GFP-2N4R+TN2 co-expressing cells showed total MT levels below those of non-transfected cells. TN1.5M and TN2 showed more efficient inhibitions than any other constructs. (*Continued*)

B

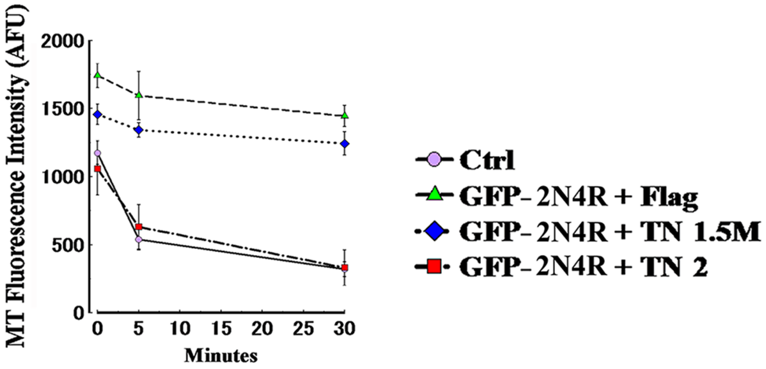

C

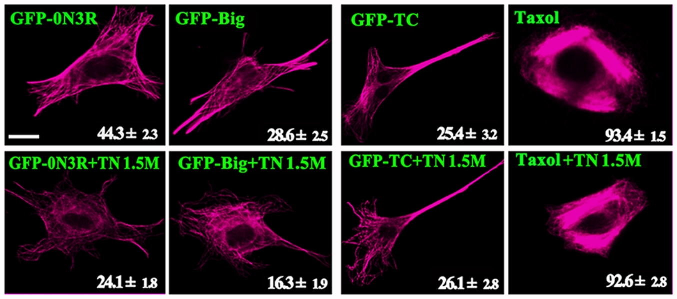

Supplementary Figure S5: (*Continued*) Selection of the MT bundling inhibitor. B. To exclude the construct with non-specific effects the stability of MTs were examined in GFP-2N4R+TN1.5M and GFP-2N4R+TN2 co-expressing cells by nocodazole treatment. The total MT levels at the indicated time points after beginning of the treatment were assessed and plotted. Note that GFP-2N4R+TN1.5M and GFP-2N4R+Flag showed similar monophasic curves with only slight reductions, while GFP-2N4R+TN2 and non-transfected control showed similar biphasic time dependent reduction curves. The latter suggests that TN2 has more than bundle inhibitory effects under the presence of full length tau. **C.** Effects of TN1.5M on three types of MT bundles. The tau N-terminus domain dependent MT bundling was induced by the expression of GFP-0N3R or GFP-Big while tau N-terminus domain independent bundling was induced by GFP-TC expression or taxol treatment (upper panels). In these conditions TN1.5M was further expressed (lower panels) and the bundle inhibitory effects were assessed as in (A). Only on tau N-terminus domain dependent MT bundles TN-1.5M showed significant bundle inhibitory effects (Student's *t*-test, $P < 0.01$) (40<cells were analyzed, n=3). Bar, 10 µm.

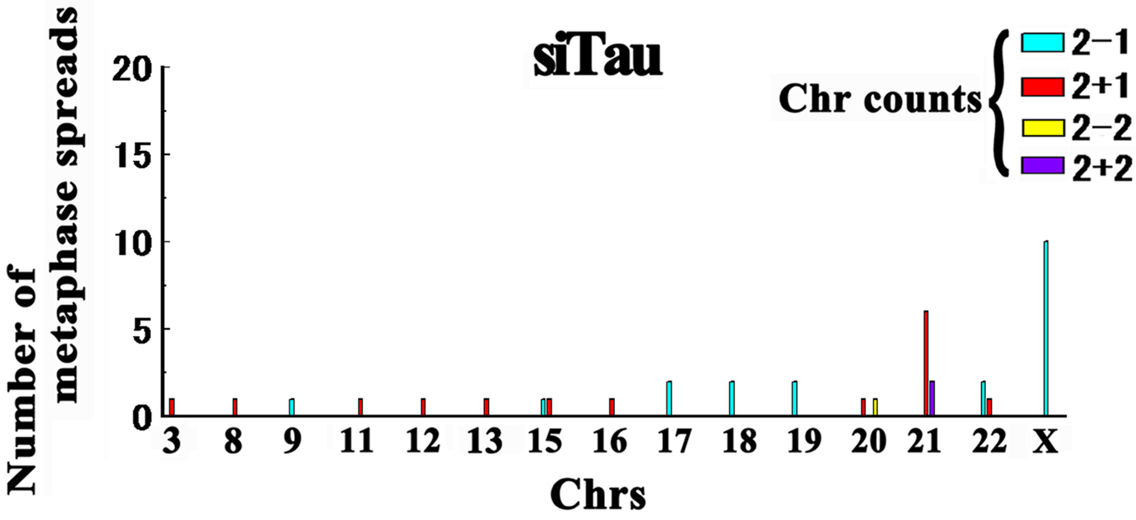

Supplementary Figure S6: Karyotyping of tau-KD HMECs. We karyotyped 127 metaphases of tau-KD cells and of the 125 diploid cells observed, 103 were normal and 22 exhibited aneuploidy (18%). The other 2 cells were tetraploid. We detected a peak (10 cells; 8%) indicating a loss of one X Chr. A second Chr 21 triplication peak was also detected (6 cells; 5%). Yellow bar: loss of two Chrs.

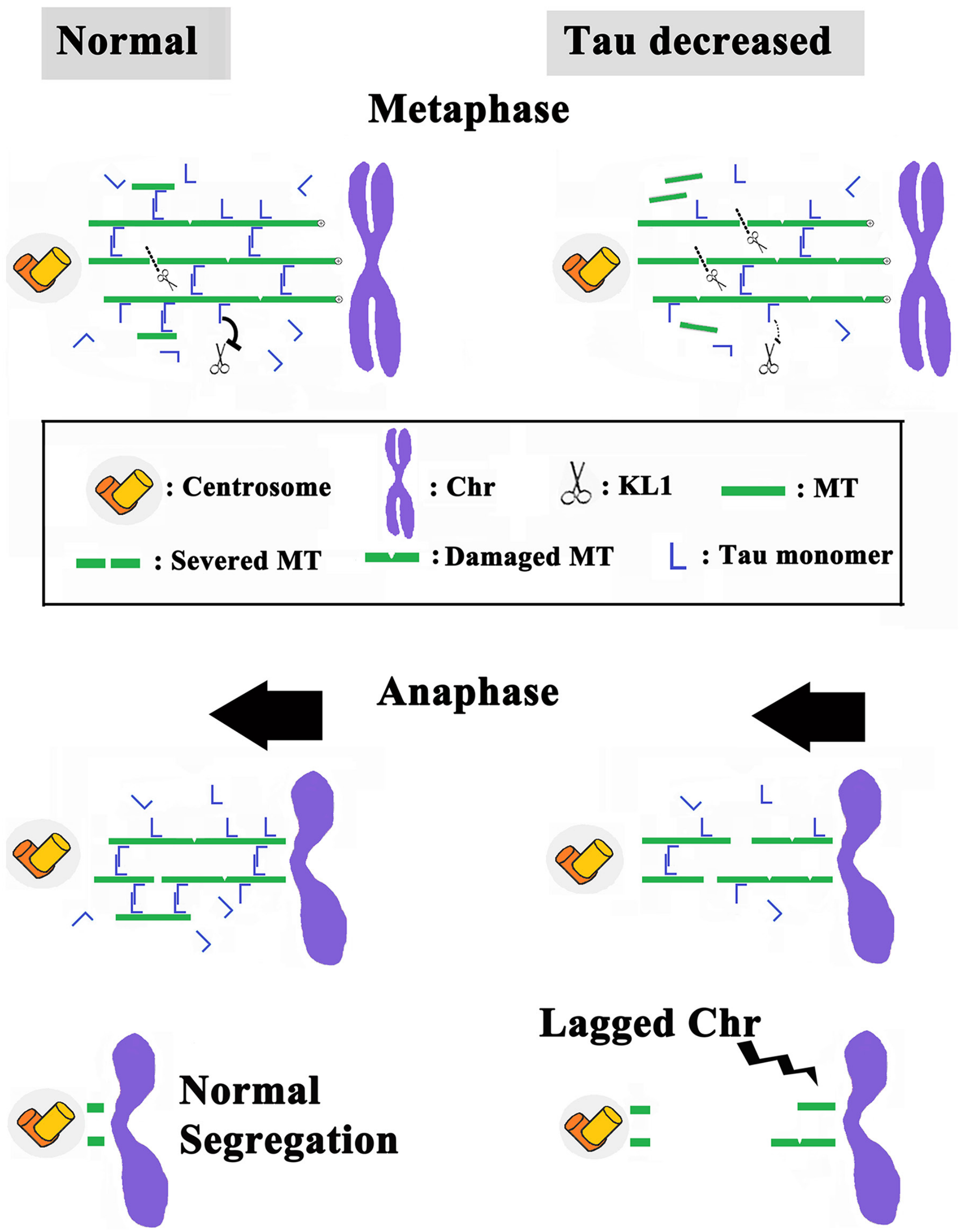

Supplementary Figure S7: Whole view of the hypothesis. Left: During normal state metaphase KL1 contributes to sever MTs in the spindle pole region and the produced short MTs are incorporated into the Kt fiber as intermingled MTs to strengthen the MT cohesiveness. The transient tau homodimer formation between MTs may be involved in this process. Too much severing is inhibited by direct inhibitory effects of tau on the surface of MT lattice (black inhibition mark) and even severed MTs keep the continuity and tension through the cross bridges between two different MTs within the Kt fiber. Tau may contribute to the formation of these cross bridges. During anaphase even though there is a decrease in the Kt-MT numbers, the severed MT or the intermediate MT damages will not cause segregation error of chromatids due to the reinforcement of MT cohesiveness by cross bridges. Right: In tau-decreased metaphase relative excess of KL1 activity may cause too much severing events. The generated short MTs however will not be efficiently incorporated into Kt fibers due to the shortage of tau. The SAC-mediated efficient detection of discontinuity or of loss of tension in Kt fibers and following recovery of connection may keep the normal morphology of Chr alignment in the spindle equator. However, simultaneously there may be increased number of the intermediate damages which would escape from SAC-mediated surveillance system. During anaphase, along with the decrease in the number of Kt-MT per fiber, the severed MT may cause discontinuity of the fiber due to the shortage of tau-mediated cross bridges. Too many intermediate MT damages also spur on the risk of the discontinuity. The probability of Chr nondisjunction or segregation error will be enhanced and that may be manifested as the lagged Chrs or Chr bridges.
